# Reconstructing the Population Genetic History of the Caribbean

**DOI:** 10.1371/journal.pgen.1003925

**Published:** 2013-11-14

**Authors:** Andrés Moreno-Estrada, Simon Gravel, Fouad Zakharia, Jacob L. McCauley, Jake K. Byrnes, Christopher R. Gignoux, Patricia A. Ortiz-Tello, Ricardo J. Martínez, Dale J. Hedges, Richard W. Morris, Celeste Eng, Karla Sandoval, Suehelay Acevedo-Acevedo, Paul J. Norman, Zulay Layrisse, Peter Parham, Juan Carlos Martínez-Cruzado, Esteban González Burchard, Michael L. Cuccaro, Eden R. Martin, Carlos D. Bustamante

**Affiliations:** 1 Department of Genetics, Stanford University School of Medicine, Stanford, California, United States of America; 2 Department of Human Genetics and Genome Quebec Innovation Centre, McGill University, Montreal, Québec, Canada; 3 Center for Genetic Epidemiology and Statistical Genetics, John P. Hussman Institute for Human Genomics, University of Miami Miller School of Medicine, Miami, Florida, United States of America; 4 Ancestry.com DNA, LLC, San Francisco, California, United States of America; 5 Department of Bioengineering and Therapeutic Sciences, University of California San Francisco, California, United States of America; 6 Department of Biology, University of Puerto Rico at Mayaguez, Mayaguez, Puerto Rico; 7 Department of Structural Biology, Stanford University School of Medicine, Stanford, California, United States of America; 8 Center of Experimental Medicine “Miguel Layrisse”, IVIC, Caracas, Venezuela; Universidade Federal de Minas Gerais, Brazil

## Abstract

The Caribbean basin is home to some of the most complex interactions in recent history among previously diverged human populations. Here, we investigate the population genetic history of this region by characterizing patterns of genome-wide variation among 330 individuals from three of the Greater Antilles (Cuba, Puerto Rico, Hispaniola), two mainland (Honduras, Colombia), and three Native South American (Yukpa, Bari, and Warao) populations. We combine these data with a unique database of genomic variation in over 3,000 individuals from diverse European, African, and Native American populations. We use local ancestry inference and tract length distributions to test different demographic scenarios for the pre- and post-colonial history of the region. We develop a novel ancestry-specific PCA (ASPCA) method to reconstruct the sub-continental origin of Native American, European, and African haplotypes from admixed genomes. We find that the most likely source of the indigenous ancestry in Caribbean islanders is a Native South American component shared among inland Amazonian tribes, Central America, and the Yucatan peninsula, suggesting extensive gene flow across the Caribbean in pre-Columbian times. We find evidence of two pulses of African migration. The first pulse—which today is reflected by shorter, older ancestry tracts—consists of a genetic component more similar to coastal West African regions involved in early stages of the trans-Atlantic slave trade. The second pulse—reflected by longer, younger tracts—is more similar to present-day West-Central African populations, supporting historical records of later transatlantic deportation. Surprisingly, we also identify a Latino-specific European component that has significantly diverged from its parental Iberian source populations, presumably as a result of small European founder population size. We demonstrate that the ancestral components in admixed genomes can be traced back to distinct sub-continental source populations with far greater resolution than previously thought, even when limited pre-Columbian Caribbean haplotypes have survived.

## Author Summary

Latinos are often regarded as a single heterogeneous group, whose complex variation is not fully appreciated in several social, demographic, and biomedical contexts. By making use of genomic data, we characterize ancestral components of Caribbean populations on a sub-continental level and unveil fine-scale patterns of population structure distinguishing insular from mainland Caribbean populations as well as from other Hispanic/Latino groups. We provide genetic evidence for an inland South American origin of the Native American component in island populations and for extensive pre-Columbian gene flow across the Caribbean basin. The Caribbean-derived European component shows significant differentiation from parental Iberian populations, presumably as a result of founder effects during the colonization of the New World. Based on demographic models, we reconstruct the complex population history of the Caribbean since the onset of continental admixture. We find that insular populations are best modeled as mixtures absorbing two pulses of African migrants, coinciding with the early and maximum activity stages of the transatlantic slave trade. These two pulses appear to have originated in different regions within West Africa, imprinting two distinguishable signatures on present-day Afro-Caribbean genomes and shedding light on the genetic impact of the slave trade in the Caribbean.

## Introduction

Genomic characterization of diverse human populations is critical for enabling multi-ethnic genome-wide studies of complex traits [1]. Genome-wide data also affords reconstruction of population history at finer scales, shedding light on evolutionary processes shaping the genetic composition of peoples with complex demographic histories. This genetic reconstruction is especially relevant in recently admixed populations from the Americas. Native peoples throughout the American continent experienced a dramatic demographic change triggered by the arrival of Europeans and the subsequent African slave trade. Important progress has been made to characterize genome-wide patterns of these three continental-level ancestral components in admixed populations from the continental landmass [2] and other Hispanic/Latino populations [3], including recent genotyping and sequencing studies involving Puerto Rican samples [4], [5], [6]. However, no genomic survey has focused on multiple populations of Caribbean descent, and critical questions remain regarding their recent demographic history and fine-scale population structure. Several factors distinguish the Antilles and the broader Caribbean basin from the rest of North, Central, and South America, resulting in a unique territory with particular dynamics impacting each of its ancestral components.

First, native pre-Columbian populations suffered dramatic population bottlenecks soon after contact. This poses a challenge for reconstructing population genetic history because extant admixed populations have retained a limited proportion of the native genetic lineages [7]. Second, it is widely documented that the initial encounter between Europeans and Native Americans, such as the first voyages of Columbus, took place in the Caribbean before involving mainland populations. However it remains unclear whether the earlier onset of admixture in the Caribbean translates into substantial differences in the European genetic component of present-day admixed Caribbean genomes, compared to other Hispanic/Latino populations impacted by later, and probably more numerous, waves of European migrants. Third, the Antilles and surrounding mainland of the Caribbean were the initial destination for much of the trans-Atlantic slave trade, resulting in admixed populations with higher levels of African ancestry compared to most inland populations across the continent. However, the sub-continental origins of African populations that contributed to present-day Caribbean genomes remain greatly under-characterized.

Disentangling the origin and interplay among ancestral components during the process of admixture enhances our knowledge of Caribbean populations and populations of Caribbean descent, informing the design of next-generation medical genomic studies involving these groups. Here, we present SNP array data for 251 individuals of Caribbean descent sampled in South Florida using a parent-offspring trio design and 79 native Venezuelans sampled along the Caribbean coast. The family-based samples include individuals with grandparents of either Cuban, Haitian, Dominican, Puerto Rican, Colombian, or Honduran descent. The 79 native Venezuelan samples are of Yukpa, Warao, and Bari tribal affiliation. We construct a unique database which includes public and data access committee-controlled data on genomic variation from over 3,000 individuals including HapMap [8], 1000 Genomes [6], and POPRES [9] populations, and African [10] and Native American [11] SNP data from diverse sub-continental populations employed as reference panels. We apply admixture deconvolution methods and develop a novel ancestry-specific PCA method (ASPCA) to infer the sub-continental origin of haplotypes along the genome, yielding a finer-resolution picture of the ancestral components of present-day Caribbean and surrounding mainland populations. Additionally, by analyzing the tract length distribution of genomic segments attributable to distinct ancestries, we test demographic models of the recent population history of the Greater Antilles and mainland populations since the onset of inter-continental admixture.

## Results

### Population structure of the Caribbean

**Figure 1:**
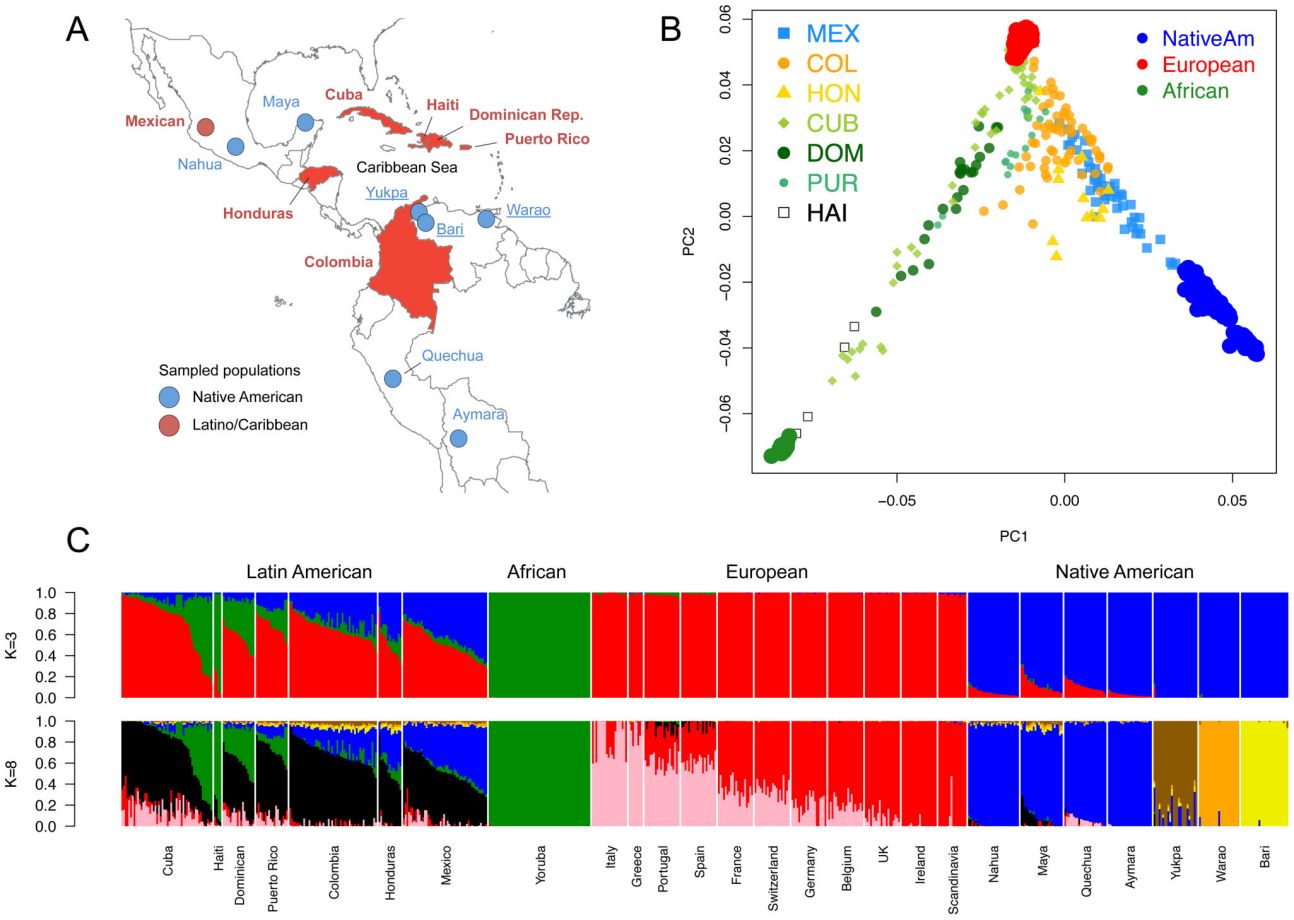
Population structure of Caribbean and neighboring populations. A) Areas in red indicate countries of origin of newly genotyped admixed population samples and blue circles indicate new Venezuelan (underlined) and other previously published Native American samples. B) Principal Component Analysis and C) *ADMIXTURE* [12] clustering analysis using the high-density dataset containing approximately 390 K autosomal SNP loci in common across admixed and reference panel populations. Unsupervised models assuming *K* = 3 and *K* = 8 ancestral clusters are shown. At *K* = 3, Caribbean admixed populations show extensive variation in continental ancestry proportions among and within groups. At *K* = 8, sub-continental components show differential proportions in recently admixed individuals. A Latino-specific European component accounts for the majority of the European ancestry among Caribbean Latinos and is exclusively shared with Iberian populations within Europe. Notably, this component is different from the two main gradients of ancestry differentiating southern from northern Europeans. Native Venezuelan components are present in higher proportions in admixed Colombians, Hondurans, and native Mayans.

To characterize population structure across the Antilles and neighboring mainland populations, we combined our genotype data for the six Latino populations with continental population samples from western Africa, Europe, and the Americas, as well as additional admixed Latino populations (see Table S1). To maximize SNP density, we initially restricted our reference panels to representative subsets of populations with available Affymetrix SNP array data (Figure 1A). Using a common set of ∼390 K SNPs, we applied both principal component analysis (PCA) and an unsupervised clustering algorithm, ADMIXTURE [12], to explore patterns of population structure. Figure 1B shows the distribution in PCA space of each individual, recapitulating clustering patterns previously observed in Hispanic/Latino populations [3]: Mexicans cluster largely between European and Native American components, Colombians and Puerto Ricans show three-way admixture, and Dominicans principally cluster between the African and European components. Ours is the first study to characterize genomic patterns of variation from (1) Hondurans, which we show have a higher proportion of African ancestry than Mexicans, (2) Cubans, which show extreme variation in ancestry proportions ranging from 2% to 78% West African ancestry, and (3) Haitians, which showed the largest average proportion of West African ancestry (84%). Additional clustering patterns obtained from higher PCs are shown in Figure S1.

We used the program ADMIXTURE to fit a model of admixture in which an individual's genome is composed of sites from up to *K* ancestral populations. We explored *K* = 2 through 15 ancestral populations (Figure S2) to investigate how assumptions regarding *K* impact the inference of population structure. Assuming a *K* = 3 admixture model, population admixture patterns are driven by continental reference samples with no continental subdivision (Figure 1C, top panel). However, higher *K*s show substantial substructure in all three continental components. Log likelihoods for successively increasing levels of *K* continue to increase substantially as *K* increases (Figure S3a), which is not unexpected since higher values of *K* add more parameters to the model (thereby improving the fit). Using cross-validation we found that *K* = 7 and *K* = 8 have the lowest predicted error (Figure S3b); thus, we focused on these two models.

The first sub-continental components that emerge are represented by South American population isolates, namely the three Venezuelan tribes of Yukpa, Warao, and Bari. At higher-order *K*s, we recapitulate the well-documented North-to-South American axis of clinal genetic variation described by us [13] and others [11], [14], as Mesoamerican (Maya/Nahua) and Andean (Quechua/Aymara) populations are assigned to different clusters (Figure S2). Interestingly, Mayans are the only group showing substantially higher contributions from the native Venezuelan components (Figure 1C, bottom panel). Both Mesoamerican and Andean Native American samples contain considerable amounts of European ancestry, due to post-Columbian admixture. Above *K* = 7, we observe a North-to-South European differentiation, which is consistent with previous analyses [15], [16]. Surprisingly, we observe another European-specific component emerge as early as *K* = 5 and remain constant through *K* = 15 (Figure S2). This component accounts for the majority of the Caribbean Latinos' European ancestry, and it only appears in Mediterranean populations, including Italy, Greece, Portugal, and Spain at intermediate proportions. Throughout this paper, we refer to this component as the “Latino European” component, and it can be seen clearly in Figure 1C (“black” bars represent the Latino European component, “Red” bars represent the “Northern European”, and pink the “Mediterranean” or “Southern European” component). At *K* = 8, when the clinal gradient of differentiation between Southern and Northern Europeans appears, the Latino European component is seen only in low proportions in individuals from Portugal and Spain, whereas it is the major European component among Latinos (Figure 1C, bottom panel).

To identify possible sex-biased gene flow in Caribbean populations, we compared the ancestry proportions of the X chromosome vs. the autosomes in each population. We observe a significant skew towards a higher proportion of Native American ancestry on the X chromosome than on the autosomes (*p*-value<10^−5^, Figure S4), consistent with previous reports on Hispanic/Latino populations [3]. Interestingly, whereas some insular populations such as Cubans and Puerto Ricans also showed a significant increase of African ancestry on the X chromosome (*p*-value<0.01), the average difference in mainland populations was not significant (*p*-value>0.05, Figure S4). Overall, we find evidence of a high Native American, and to a lesser extent African, female contribution in Caribbean populations.

Additionally, our data show a strong signature of assortative mating based on genetic ancestry among Caribbean Latinos, as suggested by previous studies [17]. In particular, we see a strong correlation between maternal and paternal ancestry proportions (Figure S5). To assess significance, we compared correlation of ancestry assignments among parent pairs to 100,000 permuted male-female pairs for each continental ancestry. All *p*-values were highly significant (*p*<0.00001, Table S2). It should be noted that these tests are not independent since the three components of ancestry by definition must sum to one. Further, apparent assortative mating could be due to random mating within structured sub-populations. To control for this, we performed permutations within countries of origin, and found significant correlations among individuals from every single population (*p*-value<0.05), except for Haiti. Although Haitians do show the same trend, with only two parent pairs, it is nearly impossible to assess significance (Table S2).

### Demographic inference since the onset of admixture

**Figure 2:**
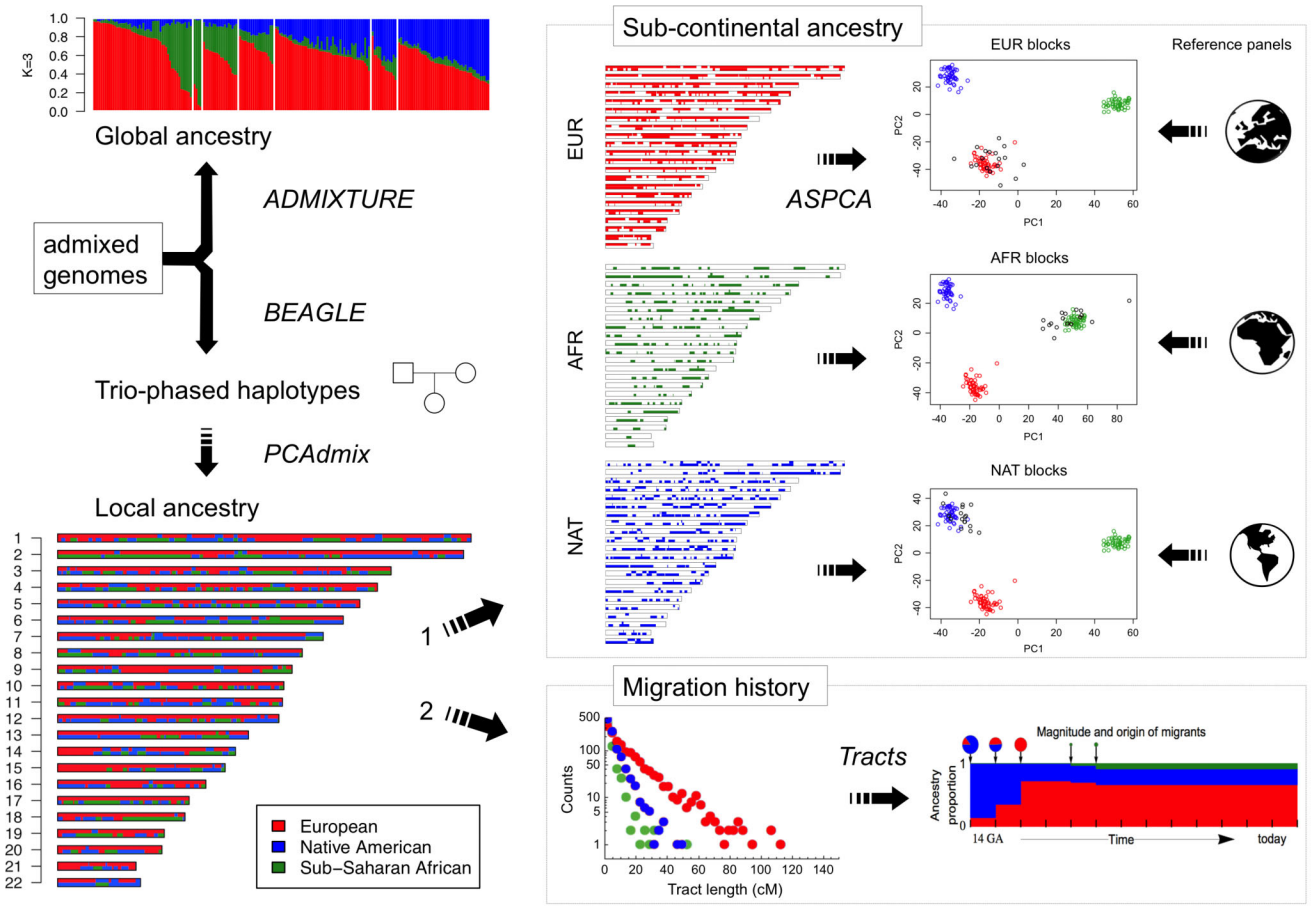
Diagram of the analytical strategy used for reconstructing migration history and sub-continental ancestry in admixed genomes. The starting point consists of genome-wide SNP data from family trios. Unrelated individuals are used to estimate global ancestry proportions with ADMIXTURE, whereas full trios are selected for BEAGLE phasing and PCA-based local ancestry estimation using continental reference samples. From here, two orthogonal analyses are performed: 1) Ancestry-specific regions of the genome are masked to separately apply PCA to European, African, and Native American haplotypes combined with large sub-continental reference panels of putative ancestral populations. We refer to this methodology as ancestry-specific PCA (ASPCA) and the code is packaged into the software *PCAmask*. 2) Continental-level local ancestry calls are used to estimate the tract length distribution per ancestry and population, which is then leveraged to test different demographic models of migration using *Tracts* software.

An overview of our analytic strategy for characterizing admixed genomes is presented in Figure 2. Due to meiotic recombination, the correlation in ancestry among founder chromosomes is broken down over time. As a consequence, the length of tracts assigned to distinct ancestries in admixed genomes is informative of the time and mode of migration [18]. To explore the population genetic history of the Caribbean since European colonization, we considered the length distribution of continuous ancestry tracts in each of the six population samples. First, we estimated local ancestry along the genome using an updated version of PCAdmix [19] which was trained using trio-phased data from the admixed individuals and three continental reference populations. Next, we characterized the length distribution of unbroken African, European, and Native American ancestry tracts along each chromosome for each population. Finally, we applied the extended space Markov model implemented in *Tracts* [20] to compare the observed data with predictions from different demographic models considering various migration scenarios.

**Figure 3:**
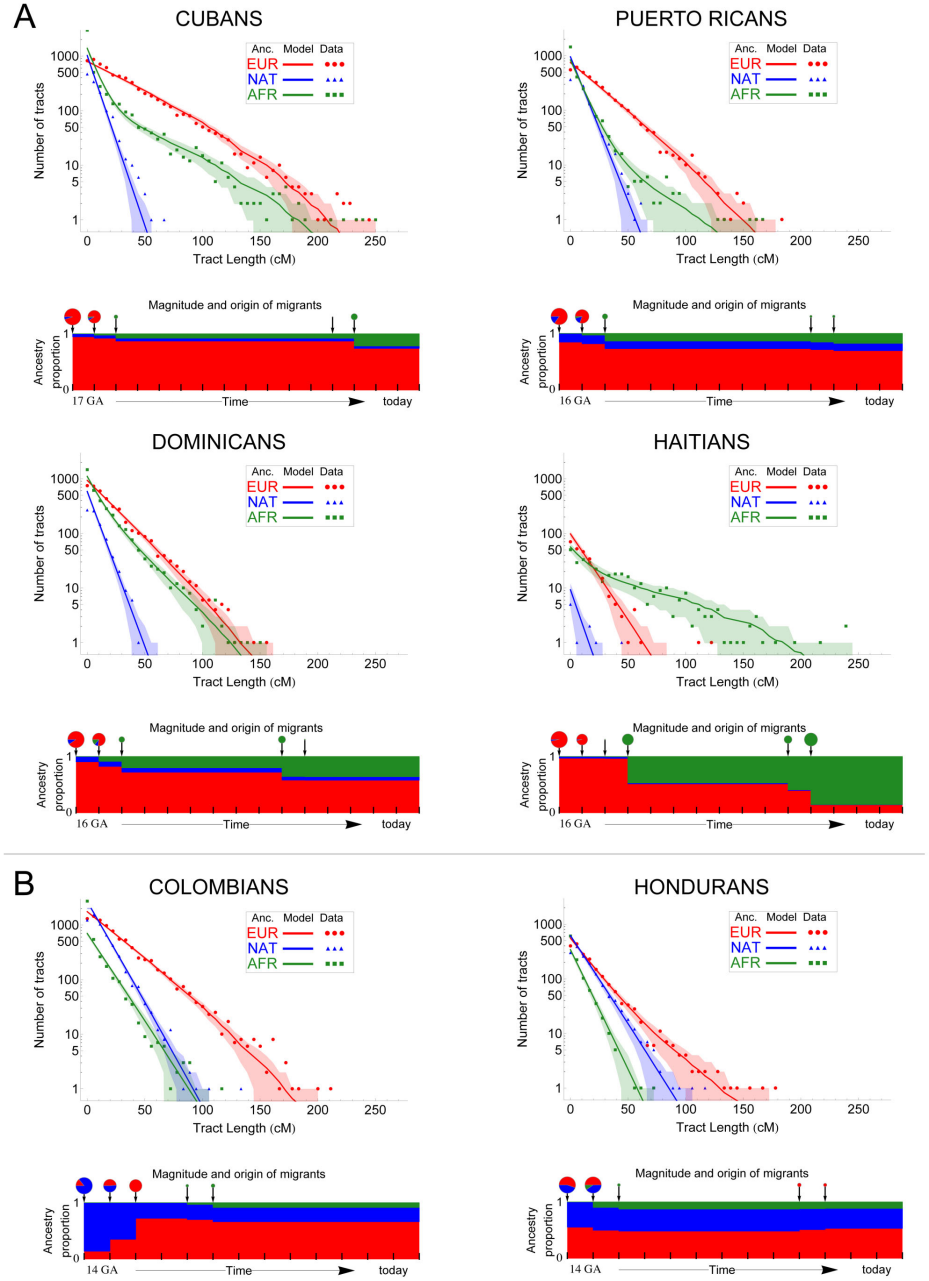
Demographic reconstruction since the onset of admixture in the Caribbean. We used the length distribution of ancestry tracts within each population from A) insular and B) mainland Caribbean countries of origin. Scatter data points represent the observed distribution of ancestry tracts, and solid-colored lines represent the distribution from the model, with shaded areas indicating 68.3% confidence intervals. We used Markov models implemented in *Tracts* to test different demographic models for best fitting the observed data. Insular populations are best modeled when allowing for a second pulse of African ancestry, and mainland populations when a second pulse of European ancestry is allowed. Admixture time estimates (in number of generations ago), migration events, volume of migrants, and ancestry proportions over time are given for each population under the best-fitting model. The estimated age for the onset of admixture among insular populations is consistently older (i.e., 16–17) compared to that among mainland populations (i.e., 14).

The simplest model considers a single pulse of migration from each source population, allowing the admixture process to begin with Native American and European chromosomes, followed by the introduction of African chromosomes. In such a scenario, each population contributes migrants at a discrete period in time, and the average length of ancestry tracts is expected to decrease with time after admixture, resulting in an exponential decay in the abundance of tracts as a function of tract length. Alternative models include a second pulse of either European or African segments migrating into the already-admixed gene pool. Allowing for continuous or repeated migration typically results in a concave log-scale distribution, caused by the increase of longer tracts after the second migration event. Table 1 and Figure 3 summarize the results of the best-fitting migration models for each population based on Bayesian Information Criterion (BIC) comparisons, and Figure S6 shows the full results of all models tested. We observed that multiple pulses of admixture exhibited a better BIC in all cases.

**Table 1:** Models of Migration into the Caribbean after the advent of admixture.

Admixed Population	Migration models^1^					
	EUR,NAT+AFR		EUR,NAT+AFR+EUR		EUR,NAT+AFR+AFR	
	Log Likelihood	Time (G)^2^	Log Likelihood	Time (G)^2^	Log Likelihood	Time (G)^2^
COL	−255.33	13	**−246.80**	**14**	−247.68	13
HON	−153.24	13	**−139.22**	**14**	−156.03	13
CUB	−506.43	19	−497.62	21	**−326.12**	**17**
DOM	−189.39	17	−189.33	17	**−170.14**	**16**
HAI	−122.73	11	−121.91	12	**−119.10**	**16**
PUR	−222.82	17	−204.23	17	**−176.17**	**16**

1 Three migration models were tested for each admixed population: a simple model of single pulses of migrants from each source population, beginning with Europeans and Native Americans at T_1_ followed by African migrants at T_2_ (EUR,NAT+AFR); the simple model followed by an additional pulse of European migrants (EUR,NAT+AFR+EUR); the simple model followed by an additional pulse of African migrants (EUR,NAT+AFR+AFR). Log likelihoods given either model were compared and we present the model with the best Bayesian Information Criterion (log likelihood values in bold).

2 The maximum likelihood estimate of time since admixture initially began. We assume prior migration between the populations was zero. Time since migration began is indicated in generations.

The best-fit model for Colombians and Hondurans involves admixture between Native Americans and Europeans starting 14 generations ago, followed by a second pulse of European ancestry starting 12 and 5 generations ago, respectively. Of note is that between the first and second pulse of migration in Colombians, the proportion of European ancestry increased from 12.5% to 75% in two generations, implying that the European segments in today's Colombians date back to European gene flow happening in a short period of time; thus, tracing their ancestry to a smaller number of European founders compared to other Latino populations.

In contrast with mainland population samples, the best-fit model for all four populations from the Caribbean islands involves older time estimates of the initial contact between Native Americans and Europeans. Namely, 17 generations ago for Cubans and 16 generations ago for Puerto Ricans, Dominicans, and Haitians. Historical records state that the first European colonies in the Antilles were established soon after the initial contact in 1492 [21]; that is, ∼500 years ago or 16.6 generations ago (considering 30 years per generation [22]), in excellent agreement with our time estimates. Another major distinction between mainland and Caribbean populations is that the best model for each of the latter involves a second pulse of African ancestry, occurring seven to five generations ago, with higher migration rates in Haitians and Dominicans, followed by Cubans and Puerto Ricans.

### Sub-continental ancestry of admixed genomes

The genomes of admixed populations contain information about both continental and sub-continental genetic ancestry. To explore within-continent population structure, we performed PCA on genomic segments assigned to Native American, African, or European ancestry. Because the masking out of the other ancestries results in large amounts of missing data, we implemented a novel variation of PCA that allows us to perform the analysis on the remaining sites alone. Throughout this paper, we refer to this approach as ancestry-specific PCA (ASPCA), and the mathematical details are described in Text S1. We applied this methodology for analyzing phased genomic segments of inferred Native American, European, and African continental ancestry together with sub-continental reference panels of parental populations (see diagram in Figure 2). Our implementation is analogous to the subspace PCA (ssPCA) approach by Johnson et al. [23], but it can take advantage of phased data, allowing us to include segments of the genome that are heterozygous for ancestry. In the presence of recent admixture, chromosomal ancestry breakpoints dramatically reduce the proportion of the genome that is homozygous for a given ancestry. Therefore, relying on genotypes and restricting to loci estimated to have two copies of a certain ancestry could severely compromise the resolution of the analysis of admixed genomes. Our haplotype-based implementation of the algorithm is packaged into the software *PCAmask* and is available at http://bustamantelab.stanford.edu. Details on the samples used are available in Materials and Methods and in Text S1.

### Native American ancestral components

Our initial structure analysis was based on our high-density dataset (i.e., ∼390 K SNPs, see Table S1), and was thus limited to ancestral populations with available Affymetrix SNP array data (i.e., two Mesoamerican, two Andean, and three Venezuelan native populations). To explore possible relationships with additional Native American populations, we expanded our reference panel by combining our data with Illumina 650 K data for 493 individuals from 52 indigenous groups from throughout the Americas [11]. Although this analysis has fewer SNPs (i.e., ∼30 K SNPs), it allows us to resolve within-continent population structure around the Caribbean in much greater geographic detail.

**Figure 4:**
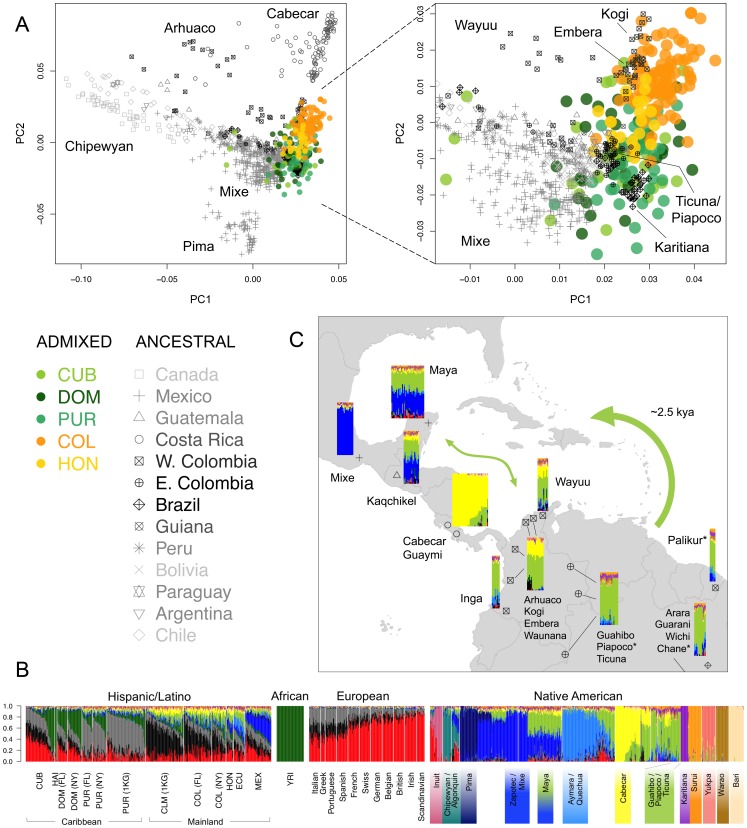
Sub-continental origin of Native American components in the Caribbean. A) Ancestry-specific PCA analysis restricted to Native American segments from admixed Caribbean individuals (colored circles) and a reference panel of indigenous populations (gray symbols) from [11], grouped by sampling location. Darker symbols denote countries of origin with populations clustering closer to our Caribbean samples. Indigenous Colombian populations were classified into East and West of the Andes to ease the interpretation of their differential clustering in ASPCA. Population labels are shown for samples defining PC axes and representative clusters within locations. B) ADMIXTURE model for *K* = 16 ancestral clusters considering additional Latino samples, a representative subset of African and European source populations, and 52 Native American populations from [11], plus three additional Native Venezuelan tribes genotyped for this project. Vertical thin bars represent individuals and white spaces separate populations. Native American populations from [11] are grouped according to linguistic families reported therein. Labels are shown for the populations representing the 12 Native American clusters identified at *K* = 16. Clusters involving multiple populations are identified by those with the highest membership values. C) Map showing the major indigenous components shared across the Caribbean basin as revealed by ADMIXTURE at *K* = 16 from B). Namely, Mesoamerican (blue), Chibchan (yellow), and South American (green). Colored bars represent individuals and their approximate sampling locations. Bars pooling genetically similar individuals from more than one population are plotted from left to right following north to south coordinates as listed by population labels. Guarani, Wichi, and Chane from north Argentina are pooled with Arara but only the location of the latter is shown to allow us to provide a zoomed view of the Caribbean region (see [11] for the full map of sampling locations). The thick arrow represents schematically the most accepted origin of the Arawak expansion from South America into the Great Antilles around 2,500 years ago according to linguistic and archaeological evidence [30]. Asterisks next to population labels denote Arawakan populations included in our reference panel. The thin arrow indicates gene flow between South America and Mesoamerica, possibly following a coastal or maritime route, accounting for the Mayan mixture and supporting pre-Columbian back migrations across the Caribbean.

We applied the ASPCA approach described above to the Native American segments of admixed individuals with >3% global Native American ancestry together with the full reference panel of ancestral populations (Figure S7). ASPC1 separates the northernmost populations of the continent from the rest, while the Brazilian Surui and Central American Cabecar define the extremes of ASPC2. Most Native American haplotypes from the admixed genomes fall along this second axis of variation, forming two overlapping population clusters: one represented primarily by Colombians and Hondurans, and the other by Cubans, Dominicans, and Puerto Ricans (no Haitian haplotypes were included due to low levels of Native American ancestry). Figure 4A shows a closer view, in which Colombians and most Hondurans cluster closer to Chibchan-speaking groups from Western Colombia and Central America, including the Kogi, Embera, and Waunana. In contrast, most Caribbean islanders cluster with Amazonian groups from Eastern Colombia, Brazil, and Guiana. The closest ancestral populations include the Guahibo, Piapoco, Ticuna, Palikur, and Karitiana, among others, some of which are settled along fluvial territories of the Orinoco-Rio Negro basin. This location may have facilitated communication from the rainforest to the coast, explaining the relationship with Caribbean native components.

Interestingly, the indigenous component of insular Caribbean samples seems to be shared across the different islands, suggesting gene flow across the Caribbean basin in pre-Columbian times. To explore this possibility into more detail, we performed a model-based clustering analysis using the full reference panel of 52 Native American populations from Reich et al. [11] in addition to our three native Venezuelan populations. Individual admixture proportions from *K* = 2 through 20 are given in Figure S8. Focusing on Native American components, the first sub-continental signal (at *K* = 4) comprised a Chibchan component mainly represented by the Cabecar from Costa Rica and the Bari from Venezuela. Higher-order clusters pulled out Amazonian population isolates such as the Surui and Warao, as well as northern populations including the Eskimo-Aleut and Pima, in agreement with the outliers detected in our ASPCA analysis (Figure S7). Interestingly, from *K* = 5 through 10, the Chibchan component is shared at nearly 100% with the Yukpa sample located near the Venezuelan coast, and at nearly 20% with Mayans from the Yucatan peninsula and Guatemala (Figure S8). Higher-order clusters maintain the connection between Mayans and South American components. For example, at *K* = 16 (the model with the lowest cross-validation error; Figure S9b), an average of 35% of the genome in Mayans is shared with a mixed South American component mainly represented by the Ticuna, Piapoco, Guahibo, Arhuaco, Kogi, Embera, Palikur, and Wichi, among others (Figure 4B and C). The presence of considerable proportions of Central and South American components in the Mayan sample is indicative of possible “back” migrations from Central America and northern South America into the Yucatan peninsula, revealing active gene flow across the Caribbean, probably following a coastal or maritime route. This observation is in agreement with our ASPCA results from admixed genomes and reinforces the notion of an expansion of South American-based Native American components across the Caribbean basin.

### European ancestral components

**Figure 5:**
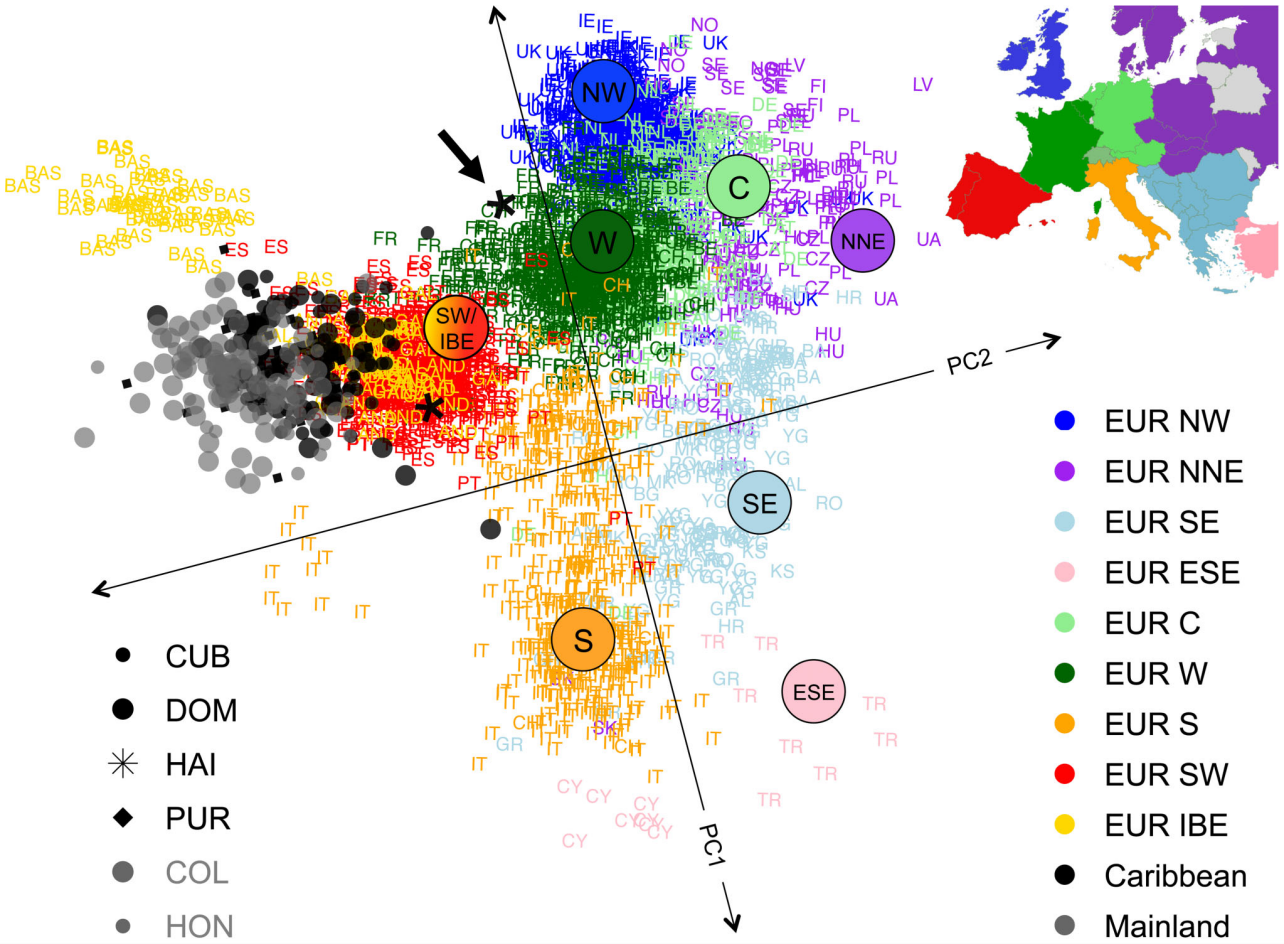
Sub-continental origin of European haplotypes derived from admixed genomes. ASPCA is applied to haploid genomes with >25% European ancestry derived from insular Caribbean (black symbols) and mainland populations (gray symbols) combined with a reference panel (colored labels) of 1,387 POPRES European samples with four grandparents from the same country [15], and 54 additional Iberian individuals (in yellow) from [24]. PC1 values have been inverted and axes rotated 16 degrees counterclockwise to approximate the geographic orientation of population samples over Europe. Population codes are detailed in Table S1 and regions within Europe are labeled as in [16]. Inset map: countries of origin for POPRES samples color-coded by region (areas not sampled in gray and Switzerland in intermediate shade of green to denote shared membership with EUR W, EUR C, and EUR S). Most Latino-derived European haplotypes cluster around the Iberian cluster. One of the two Haitian individuals included in the analysis clustered with French speaking Europeans (black arrow), in agreement with the colonial history of Haiti and illustrating the fine-scale resolution of our ASPCA approach.

We performed ASPCA analysis restricted to European segments of admixed individuals with >25% of European ancestry and a panel of European source populations, including 1,387 individuals from Europe sampled as part of the POPRES project [9], as well as additional Iberian samples from Galicia, Andalusia, and the Basque country in Spain [24]. The combined dataset included 2,882 European haplotypes and 255 haplotypes of European ancestry from the admixed populations. Figure 5 shows the first two PCs, where, as reported previously, the reference samples recapitulate a map of Europe [15], [25]. While most of the additional Iberian samples cluster together with the POPRES individuals sampled as Portuguese and Spanish, the Basques cluster separately from the centroid of most Iberian samples. The Basques are known for their historical and linguistic isolation, which could explain their genetic differentiation from the main cluster due to drift. Given the known Iberian origin of the first European settlers arriving into the Caribbean and surrounding territories of the New World, one would expect that European blocks derived from admixed Latino populations should cluster with other European haplotypes from present-day Iberians. Indeed, our Latino samples aggregate in a well-defined cluster that overlaps with the cluster of samples from the Iberian Peninsula (i.e., Portugal and Spain). However, we observed that the centroid is substantially deviated with respect to the Iberian cluster (bootstrap *p*-value<10^−4^, see Materials and Methods), suggesting the possibility of a bottleneck and drift impacting the European haplotypes of Latinos.

Importantly, when we applied ASPCA using the exact same reference panel of European samples but analyzing Mexican haplotypes of European ancestry (Moreno-Estrada, Gignoux et al., in preparation), we did not observe a deviated clustering pattern from the Iberian cluster: the effect is much weaker and not significant (bootstrap *p*-value = 0.099, see Figure S10). Furthermore, the deviation of the European segments of Mexican individuals from the distribution of the rest of Iberian samples is even smaller than the deviation of the Portuguese from the Spanish samples. We further evaluated whether the dispersion of the different subpopulations within the Caribbean cluster follow particular patterns along ASPC2, the axis driving the deviation from the Iberian centroid. We observed that Colombians and Hondurans tend to account for lower (more deviated) ASPC2 values compared to Cubans, Dominicans, and Puerto Ricans (Figure S11), suggesting a mainland versus insular population differentiation. We performed a Wilcoxon rank test to contrast ASPC2 for mainland (Colombia and Honduras) versus island (Cuba, Dominican Republic and Puerto Rico) populations, resulting in a highly significant *p*-value (1.5×10^−15^). Because >25% of European ancestry was required for inclusion in ASPCA, only two Haitian haplotypes were analyzed, and thus these were not included in the statistical analysis. Nonetheless, it is noteworthy that one of them clusters with the French, in agreement with historical and linguistic evidence regarding European settlements on the island (see arrow on Figure 5).

Among European populations, Iberians also have the highest proportion of identical by descent (IBD) segments that are shared with Latino populations, as measured by a summed pairwise IBD statistic that is informative of the total amount of shared DNA between pairs of populations (see Materials and Methods and Figure S12). To explore the distribution of IBD sharing within continental groups, we considered Caribbean Latinos and Europeans separately by summing the cumulative amount of DNA shared IBD between each pair of individuals within each group. If European segments from Latino populations derive from a reduced number of European ancestors, then IBD sharing should be higher among Caribbean individuals compared to Europeans. Indeed, we observed a higher number of pairs sharing larger total IBD segment lengths among Latino individuals than among Europeans (Figure S13). Within-population cryptic relatedness is also compatible with increased IBD sharing. However, this is more likely to occur between individuals from the same subpopulation (e.g., COL-COL) rather than individuals from geographically separated subpopulations (e.g, COL-PUR). For this reason, we repeated the analysis, excluding within-population pairs of Latino individuals, and compared the IBD distribution to that of Iberian source populations (i.e., Spanish and Portuguese). Once again, we observed an increased proportion of IBD sharing among Latinos, arguing for a shared founder effect (Figure S13).

These results are in agreement with our cluster-based analysis focused on global ancestry proportions, where the European ancestry of Latinos is dominated by a shared Latino-specific component differentiated from both southern and northern European components, although shared to some extent with Spanish and Portuguese (Figure 1C). Bottlenecked populations may exhibit differentiation from their parental gene pool due to loss of genetic diversity and stochastic shifts in allele frequencies. One way of quantifying the extent of genetic drift is to compare F_ST_ estimates among the *K* = 8 ancestral clusters from Figure 1C. In the absence of drift, we would expect the southern-derived Latino component and the southern European component to show a very low level of F_ST_. However, we observe an F_ST_ = 0.021 (Table S3). To put this into perspective, the F_ST_ of southern vs. northern Europe is F_ST_ = 0.02, meaning that the differentiation of the Latino-specific component with respect to southern Europeans is at least as high as the north-south differentiation within Europe. This observation was replicated when including additional Latino and ancestral populations (Figure S8). Given the increased number of divergent clusters, we focused on *K* = 18 through 20, in which all sub-continental European components were jointly detected. In this case, the Latino-specific component shows further fragmentation into two components: one predominantly shared among insular Caribbean samples and the other among mainland Latinos. The F_ST_ value for southern versus northern European differentiation was 0.039, while values for southern versus insular (0.041) or mainland Latinos (0.04) were slightly inflated (Table S4), supporting the notion of additional differentiation impacting the European component of present-day admixed Latinos.

### African ancestral components

The Caribbean region has a complex history of population exchange with the African continent as a result of slave trade practices during European colonialism. Its proximity to the North Atlantic Ocean facilitated nautical contact with the West African coast, increasing the exposure of the local population to slave trade routes and ultimately resulting in genetic admixture between Caribbean and African individuals. We found the proportion of African ancestry to be higher in Caribbean populations compared to those from the mainland (Figure 1C), a finding that is consistent across studies [3], [6], [26]. To explore the sub-continental composition of African segments derived from Caribbean admixed genomes, we performed ASPCA analysis on individuals with more than 25% of African ancestry using a diverse panel of African populations as potential sources (see Table S1). Our first approximation showed no dispersion of Afro-Caribbean haplotypes over PCA space. Instead, they form a relatively tight cluster that overlaps with that of the Yoruba sample from southwestern Nigeria (Figure S14). This is a plausible result, given the extensive historical record supporting a West African origin for the African lineages in the Americas.

However, according to our tract length analysis, there is strong genetic evidence for the occurrence of at least two pulses of African migrants imprinting different genomic signatures in present day admixed Caribbean populations. This result raises the question of whether both pulses involved the same source population during the admixture process. If this were the case, it would easily explain our ASPCA results, where all African haplotypes point to a single source.

**Figure 6:**
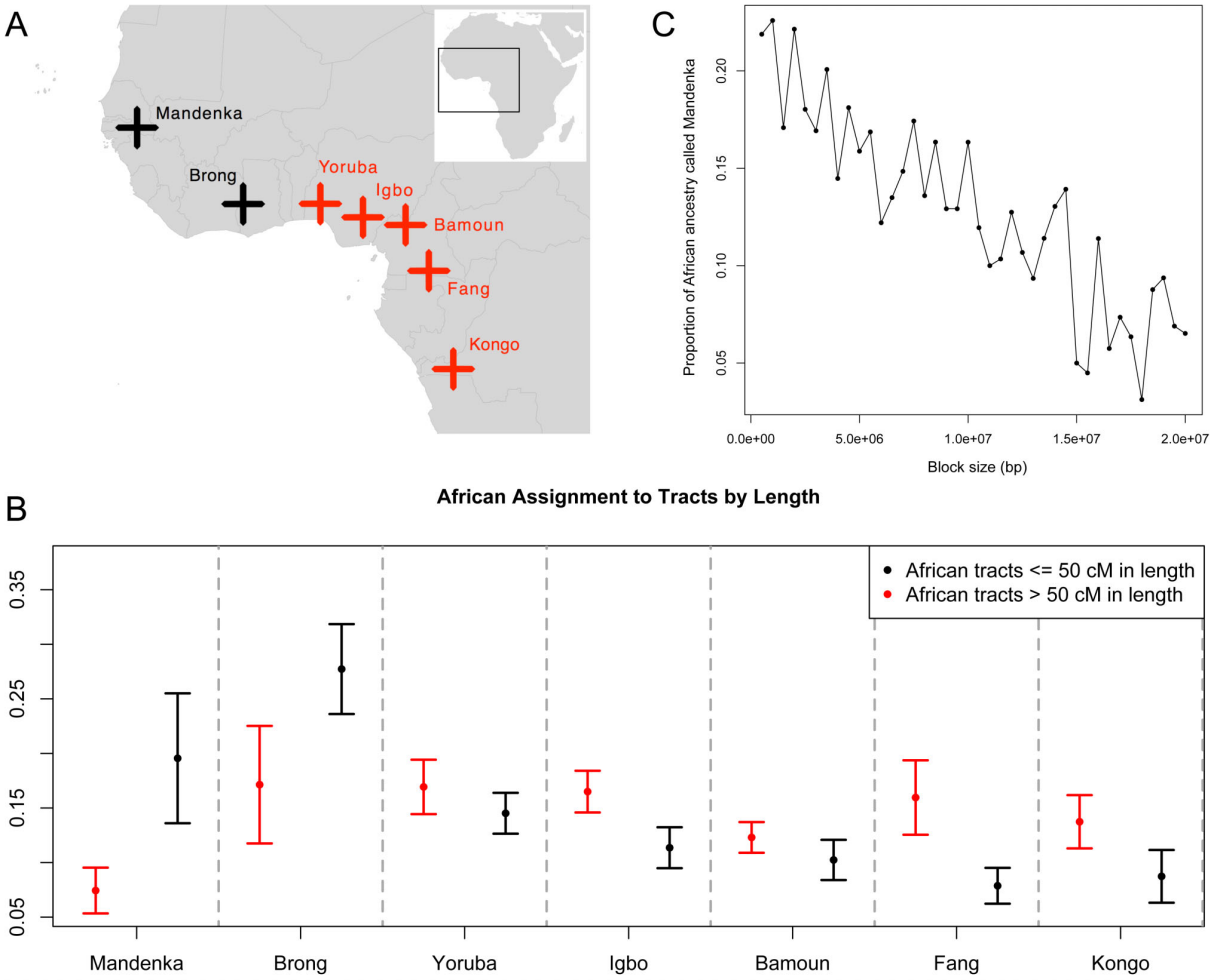
Sub-continental origin of Afro-Caribbean haplotypes of different sizes. A) Map of West Africa showing locations of reference panel populations. Samples in black are more likely to represent the origin of short ancestry tracts and those in red of long ancestry tracts, according to B) assignment probabilities for each putative ancestral population of being the source for short (<50 cM in black) and long (>50 cM in red) ancestry tracts. African ancestry tracts for Puerto Ricans are shown and results for all populations are available in Figure S16. C) Proportion of African ancestry of inferred Mandenka origin as a function of block size in the combined set of Caribbean genomes. By running *PCAdmix* within the previously inferred African segments, we obtained posterior probabilities for Mandenka versus Yoruba ancestry. Overall, we found evidence for a differential origin of the African lineages in present day Afro-Caribbean genomes, with shorter (and thus older) ancestry tracts tracing back to Far West Africa (represented by Mandenka and Brong), and longer tracts (and thus younger) tracing back to Central West Africa.

Alternatively, if more than one source were involved and if enough mixing occurred since the two pulses, it is possible that what we see in ASPCA is the midpoint of the two source populations, causing the difference to remain undetected by our standard approach (which gives a point estimate averaging the signature of all African blocks along the genome). Hence, we applied a different strategy, in which ASPCA is performed separately for short (thus older) and long (younger) ancestry tracts. For this purpose, we split the African segments of each haploid genome into two categories based on a 50-cM length cutoff and intersected the data with a reference panel of West African populations (Figure 6A). Then, for each individual, we computed assignment probabilities of coming from each of the putative parental populations based on bivariate normal distributions fitted around each PCA cluster (see Materials and Methods, Figure S15). In Figure 6B we present the scaled mean probabilities for long (>50 cM) versus short (<50 cM) African tracts in Puerto Rican individuals. The pattern that emerges reveals that African haplotypes shorter than 50 cM are more likely to have originated from populations in the coastal Northwest region, such as the Mandenka and Brong; whereas longer haplotypes show higher probabilities of coming from populations closer to the Gulf of Guinea and Equatorial West Africa, including Yoruba, Igbo, Bamoun, Fang, and Kongo (see map on Figure 6A). The significant increase in old, short Mandenka tracts when compared to longer, more recent tracts was replicated in other insular Caribbean populations, including Cubans and Dominicans. The Brong also seem to have had a greater contribution deeper in the past, not only in Puerto Ricans, but also in Dominicans, Hondurans, and to a lesser extent in Colombians. In Cubans, the trend is reversed, and the Brong seem to have contributed more to long tracts than to short ones (Figure S16).

One caveat of this analysis is that short ancestry tracts are more likely to be misassigned. To rule this out as a source of the signal, we added an intermediate block size category (>5 cM and <50 cM) and repeated the size-based ASPCA analysis. We observed that, despite the signal being somewhat weaker due to less data, a similar trend was observed after excluding extremely short tracts (Figure S16). Finally, we gathered additional evidence by running local ancestry estimation on the African blocks alone to distinguish Mandenka vs. Yoruba ancestry tracts (see Materials and Methods). We then binned all segments of inferred Mandenka ancestry into different block sizes and observed that the proportion of the African ancestry called Mandenka is higher within shorter block sizes and decreases as block size increases (Figure 6C). This result gives additional support for the differential origin of African segments and argues that the signal is not driven by the shortest genomic segments alone; rather, the signal is characterized by a progressive decay of haplotype length from older migrations, as younger segments (of different ancestry) account for the majority of longer African tracts in Caribbean genomes.

## Discussion

### Models of admixture for Caribbean and mainland populations

Our results reveal consistent differences in the admixture processes occurring on Caribbean islands as compared to neighboring mainland populations. First, admixture timing estimates are consistently different between these two groups, with admixture starting around 16–17 generations ago in the islands and 14 generations ago in mainland populations. Second, in the Caribbean, we find evidence of a single pulse of Native American ancestry into admixed populations. Since Native American tracts are shorter, on average, than tracts of any other ancestry (and therefore older), this suggests an initial contribution at the time of European contact with limited subsequent contribution, consistent with the rapid decimation of the native population. Mainland populations from Colombia and Honduras, on the other hand, exhibit longer Native American tracts and are best fit by a model with a greater contribution of Native American ancestry. Third, Caribbean populations show evidence of limited number of European pulse events, suggesting a limited number of founders contributed disproportionally to the present day population. Continental populations, on the other hand, show evidence of repeated migration events of European ancestry, consistent with a continuing expansion of Europeans during colonialism. Finally, our data also suggest that multiple pulses of African migration contributed significantly to genetic ancestry in the Caribbean, consistent with records of historical slave trade routes. In contrast, African ancestry tracts in mainland populations are consistent with a more limited influx of African migrants.

The abundance of historical accounts regarding European colonization of the New World facilitates the contrast between written and genetic records. Our models show remarkable agreement with historical records. The earliest European contact in the Americas dates back to 1492, involving the Caribbean island of Hispaniola (today's Dominican Republic and Haiti). First contact dates are upper bounds on the time at which demographically substantial admixture would have taken place. The fact that our admixture timing estimate (i.e., 16–17 generations ago) is so close to first contact emphasizes that the colonization proceeded rapidly, with substantial admixture taking place very quickly, as opposed to it being a more drawn out process. Later European voyages reached the coasts of Central and South America, so permanent European settlements did not occur in the mainland until the first half of the 16^th^ century, consistent with an approximate difference of two generations between the estimated onset of admixture according to our island and mainland models. Here we have focused on Colombians and Hondurans as population samples from mainland territories with coastal access to the Caribbean, but we have previously reported admixture timing estimates for Mexicans as well, starting 15 generations ago [5]. The settlement of Europeans in mainland Mexican territory is documented to have occurred between 1519 and 1521 (i.e., 27–29 years apart from the first contact in 1492 in the Caribbean); consistent with this, there is one generation between our average estimate for the onset of admixture in the Caribbean compared to our model based on Mexican data (16 vs. 15 generations, respectively).

### South American origin of indigenous components in the Caribbean

In contrast to other regions in the Americas where indigenous peoples are numerous, the genetic characterization of Native American components in the Caribbean required indirect reconstruction via genomic assembly of indigenous ancestry tracts transmitted to extant admixed individuals. By applying ancestry-specific PCA and cluster-based analyses integrating a large number of indigenous groups throughout the Americas, we found that Amazonian populations from South America show the closest relationship with Caribbean indigenous components. This was also observed in a different sample set from the 1000 Genomes Project (Gravel et al., submitted). Despite covering a large geographic area of South America (ranging from eastern Colombia to central Brazil and Guiana), most Amazonian sampled populations cluster together in PCA space, suggesting a common origin. Logical candidates for the origin of the ancestors of Caribbean populations include indigenous coastal groups south of the Lesser Antilles. Here, therefore, we have included three additional tribes from the Venezuelan coast. However, despite their closer geographic location, none of these groups primarily accounted for the indigenous ancestry of the insular Caribbean samples, pointing to an inland origin rather than a coastal one. Nonetheless, our cluster-based analysis revealed that native Venezuelan components do share membership with several Central American indigenous populations, such as the Costa Rican Cabecar, and, to a lesser extent, with Mayan groups from Guatemala and the Yucatan peninsula of present day Mexico, suggesting substantial gene flow across the Caribbean Sea in pre-Columbian times. Archaeological evidence, including the distribution of jade, obsidian, pottery, and other commodities, supports the existence of maritime-based interaction networks between central Mesoamerica, the Isthmo-Colombian area, and northern Venezuela [27]. Our results demonstrate that such long-distance negotiations were accompanied by genetic exchange between previously diverged native populations and give new insight into the dynamics between the inhabitants of the Caribbean basin prior to European contact.

In a recent genomic survey of the relationships between Native American peoples, Reich and colleagues [11] described the Chibchan speakers on both sides of the Panama isthmus as an exception to the simple model of continental colonization involving a southward expansion with sequential population splits and little subsequent gene flow. Instead, Central Americans, such as the Cabecar from Costa Rica, were modeled as a mixture of South and North American ancestry, which the authors reported as evidence for a back-migration from South into Central America. Our findings support these interpretations and also suggest a distant connection between Caribbean Mesoamerica and South American inland territories. Specifically, the fact that Mayans from the Yucatan peninsula share 35% of their genome with the Amazonian Ticuna, Guahibo, and Piapoco, and even with the more distant Paraguayan Guarani and north Argentinian Wichi, supports the expansion of an inland South American component across the Caribbean. For context, it is noteworthy that in ASPCA, the native ancestry tracts of Colombians and Hondurans cluster with geographically closer indigenous tribes, such as Chibchan speakers from western Colombia and Central America.

How do we account, then, for a shared clustering between more distant tribes, mostly of Amazonian origin, and insular Caribbean haplotypes? One possible explanation is that the fluvial nature of most of these settlements (across the Amazon and Orinoco basins) may have facilitated the movement of people to the coast, from which they migrated north through the Lesser Antilles and eventually contributed to Caribbean native components. Our results are consistent with archaeological records suggesting that the ancestors of the indigenous people that Columbus encountered might have come from populations that migrated from the Lower Orinoco Valley around 2.5 to 3 kya [28], [29], [30].

Additionally, our results align with the classification of languages spoken by pre-Columbian inhabitants of the Caribbean. The Taínos were the major group living in the Greater Antilles and surrounding islands at the moment of European contact. Taínos and insular Caribs spoke Arawakan languages [31], whose geographic distribution across northern South America resembles the distribution of the genetic component shared across multiple Amazonian individuals (Figure 4C). Arawakan-speaking groups in our reference panel include the Piapoco from eastern Colombia, the Palikur from Guiana, and the Chane from northern Argentina, all of which show primary ancestral membership to the Amazonian genetic component (Figure 4C) and cluster together with Native American haplotypes from admixed Caribbean individuals (Figure 4A), supporting a South American origin of the Arawakan expansion into the Caribbean. Although now located far from Amazonia, the Chane are believed to have historically migrated from the Amazon rainforest to the Argentinian Gran Chaco [32]. Neighboring Wichi individuals also show similar genetic memberships and ASPCA clustering patterns, despite belonging to a different linguistic family. Previous genetic studies have also pointed to a South American origin for Taínos [7], [33]. Based on mitochondrial haplogroups ascertained from pre-Columbian Taíno remains, Lalueza-Fox and colleagues [33] found that only two of the major mtDNA lineages, namely C and D, were present in their sample (N = 27). Given that high frequencies of C and D haplogroups are more common in South American populations, the authors argued for that sub-continent as the homeland of the Taíno ancestors.

Overall, our analysis of indigenous ancestry tracts from extant admixed genomes supports previous linguistic, archaeological, and ancient DNA evidence about the peopling of the Caribbean; furthermore, it points to a greater involvement of inland Amazonian populations during the last migration into the Antilles prior to European contact. Earlier migrations may have occurred (e.g., from Mesoamerica or the Florida peninsula), as pre-ceramic archaeological evidence of human presence in the Greater Antilles dates back more than 7,000 years ago [28]. However, the fact that the Amazonian component is shared among the indigenous haplotypes from different insular and continental populations supports either a single South American origin of Caribbean settlers or a major population replacement involving a more recent migration of agriculturalists from inland South America.

### Founder effect in the European lineage of admixed Latinos

We find genomic patterns compatible with the effect of a founder event in the ancestral European population of present-day admixed Latinos. Supporting evidence includes the following: 1) a Latino-specific European component revealed by clustering algorithms, which is not assigned to source populations within Europe except Spain and Portugal, and detected at lower-order clusters compared to other European and Native American sub-continental components; 2) inflated F_ST_ values between the Latino-specific and southern European components, compared to southern versus northern Europe differentiation; 3) significant deviation of the distribution of European haplotypes from the main cluster of Iberian samples in ASPCA space; and 4) increased IBD sharing among Latino individuals compared with Europeans. Additionally, a similar signature was observed in an independent dataset of Latino samples from the1000 Genomes Project using a combined approach that integrates IBD and local ancestry tracts (Gravel et al., submitted). These findings suggest that early European waves of migration into the New World involved a reduced ancestral population size, mainly composed of Iberians, bearing a subset of the diversity present within the source population and causing the derived admixed populations to diverge from current European populations. Furthermore, we find differences between mainland and insular Caribbean populations including 1) different time estimates for the onset of admixture as revealed by ancestry tract length analysis (Figure 3); 2) separate memberships in cluster-based analyses (Figure 4B, Figure S8); and 3) significantly shifted distributions of European haplotypes within the Latino cluster in ASPCA space (Figure 5, Figure S11). The fact that mainland Colombians and Hondurans show not only the highest proportions of the Latino-specific European component in ADMIXTURE but also the most extreme deviation from the Iberian cluster in ASPCA suggests stronger genetic drift in these populations, compatible with a two-stage European settlement involving insular territories at first, and mainland populations subsequently absorbing a subset of migrants from the islands.

There is documented evidence of extensive migration from the islands to the continent throughout the 16^th^ century [21]. There were only two viceroyalties of the Spanish Empire in the New World until the 18^th^ century: the Viceroyalty of New Spain (capital, Mexico City) and the Viceroyalty of Peru (capital, Lima). An additional viceroyalty in South America was created in 1717 with Bogota as capital (Viceroyalty of New Granada), promoting economic and population growth.

Interestingly, the estimated time for the second pulse of European migrants into the ancestors of present-day Colombians (i.e., 12 generations ago) coincides with the creation of the Colombian-based Viceroyalty of New Granada, accounting for the large increase (from 12.5% to 75%) of European ancestry in the model based on tract length distributions. This small contribution of European ancestry at the onset of admixture in Colombians reinforces the idea that their patterns of European diversity are heavily impacted by a reduced number of founders. In contrast, Mexican-derived European haplotypes do not appear to be impacted by founder events as much as the Caribbean populations analyzed here. A possible explanation is that present-day Mexico was the center of the wealthy Viceroyalty of New Spain, one of the largest European settlements under Spanish rule. This status ensured continuous exchange with Spain throughout colonial times, resulting in a larger ancestral population size.

### Space and time distinction of African migrations into the Caribbean

We find that populations from the insular Caribbean are best modeled as mixtures absorbing two independent waves of African migrants. Assuming a 30-year generation time [22], the estimated average of 15 generations ago for the first pulse (circa 1550) agrees with the introduction of African slaves soon after European contact in the New World. At first, local natives were used as the source of forced labor, but populations were decimated rapidly, giving rise to the four-century-long transatlantic slave trade, which is usually divided into two eras. The first one accounted for a small proportion (3–16%) of all Atlantic slave trade, whereas the second Atlantic system peaked in the last two decades of the 18^th^ century, accounting for more than half of the slave trade. This period of increased activity coincides with the estimated age of the second (and stronger) pulse of African tracts according to our model (e.g., 7 generations ago in Dominicans), pointing to the late 18^th^ century. In other words, the estimated time separation between these two pulses (i.e., 8 generations or ∼240 years) based on genetic data is in extraordinary agreement with historical records, recapitulating the span between the onset of African slave trade and its period of maximum intensity right before its rapid decline during the 19^th^ century [34].

To address the question of whether there was also a separation in space between the origins of these two pulses, we relied on the fact that chromosomes from older contributions to admixture have undergone more recombination events, thus leading to shorter continuous African ancestry tracts. By conducting two different but complementary size-based analyses restricted to genomic segments of inferred African ancestry, we provide compelling evidence that short African tracts are enriched with haplotypes from northern coastal West Africa, represented by Mandenka samples from Senegal and Brong from western Ghana, near the Ivory Coast. This is in agreement with documented deportation flows during the 15^th^–16^th^ centuries, wherein most enslaved Africans were carried off from Senegambia and departed for the Americas from the Gorée Island, near Cape Verde [34]. African slaves were obtained by European traders in ports along the West African coast, but raiding zones extended inland with the involvement of local African kingdoms. The Mandinka Kingdom of Senegambia was part of the Mali Empire, one of the most influential domains in West Africa, spreading its language, laws, and culture along the Niger River. The empire's total area included nearly all the land between the Sahara Desert and coastal forests, and by 1530 reached modern-day Ivory Coast and Ghana, possibly accounting for the shared pattern between the Mandenka and Brong with respect to the Caribbean's short ancestry tracts. While this interpretation is supported by the fact that the Mandenka and Brong are the westernmost population samples of our reference panel, the lack of additional samples from northern West Africa prevent us from determining whether this pattern is shared with other tribes as well. On the other hand, the greater affinity of the longer ancestry tracts with the rest of the African samples, which cover much of the central West African coast, is compatible with the greater involvement of such regions in the slave trade during the 18^th^ century.

The volume of captives being embarked from the bights of Benin (e.g., today's Nigeria) and Biafra (e.g., today's Cameroon) was so elevated after 1700 that part of its shore soon became known as the “Slave Coast” [34]. Population samples around this area represented in our reference panel include the Yoruba and Igbo from Nigeria, and the Bamoun and Fang from Cameroon, all of which show higher probabilities of being assigned as the source for longer African ancestry tracts in the admixed Latino groups analyzed. Together with Brazil, the Caribbean Islands were the major slave import zone during the 18^th^ century. Later deportation flows in the 19^th^ century involved ports of origin near the Congo River in West Central Africa. The closest population sample of our reference panel from this region is represented by the Kongo, which also shows higher affinity with longer ancestry tracts, compatible with a later contribution to admixture in the Caribbean. The 19^th^ century also saw the abolition of slavery in most parts of the world; however, the massive international flow of people it involved remains as one of the deepest signatures in the genomes of descendent populations. While the geographic extension of the regions of origin of African slaves brought to the Americas has been widely documented, it was unclear until now the extent to which particular sub-continental components have shaped the genomic composition of present-day Afro-Caribbean descendants. Our ancestry-specific and size-based analyses allowed us to discover that African haplotypes derived from Caribbean populations still retain a signature from the first African ancestors despite the later dominance of African influx from multiple sub-continental components.

### Conclusion

Our genome-wide dense genotyping data from six different populations of Caribbean descent, coupled with the availability of large-scale reference panels, allowed us to address long-standing questions regarding the origin and admixture history of the Caribbean Basin. The differences between insular and continental Caribbean populations underscore the importance of characterizing admixed populations at finer scales. We report ancestry-specific recent bottlenecks affecting particular Latino groups, but not others, which may have important implications in the expected relative proportion of deleterious mutations and elevated allele frequencies that can be detected via association studies in theses populations. Finally, the extensive population stratification within sub-continental components implies that medically relevant genetic variants may be geographically restricted, reinforcing the need for sequencing target populations in order to discover local variants that may only be relevant in Latino-specific association studies for disease.

## Materials and Methods

### Samples and data generation

Generated data and assembled datasets for this study are summarized in Table S1. A total of 251 individuals representing six different Caribbean-descent populations were recruited in South Florida, USA. Participants were required to have at least three grandparents from their countries of origin, thus limited ethnographic and anonymous pedigree information was collected. The majority of pedigrees (94.3%, n = 82) had four grandparents from the same country. Only 5 pedigrees (5.7%) had one grandparent from a different country. Informed consent was obtained from all participants under approval by the University of Miami Institutional Review Board (study no. 20081175). A total of 76 trios, 2 duos, and 19 parents were genotyped using Affymetrix 6.0 SNP arrays, which included: 80 Cubans, 85 Colombians, 34 Dominicans, 27 Puerto Ricans, 19 Hondurans, and 6 Haitians. Genotype data will be made available through dbGaP under the Genomic Origins and Admixture of Latinos (GOAL) study. Out of 173 founders, 18 samples were filtered from structure analyses due to cryptic relatedness as inferred by IBD>10%. Four trios were not considered for trio phasing due to an excess of Mendelian errors (>100 K), two trios were removed due to 3^rd^ or higher degree of relatedness between parents as inferred by IBD, and five trios were filtered due to cryptic relatedness between members of different trios above 10% IBD. After filtering, 65 complete trios remained for haplotype-based analyses. To study population structure and demographic patterns involving relevant ancestral populations, 79 previously collected samples from three native Venezuelan tribes were genotyped using the same array (i.e., 25 Yukpa [aka Yucpa], 29 Bari, and 25 Warao). We combined our data with publicly available genomic resources and assembled a global database incorporating genome-wide SNP array data for 3,042 individuals from which two datasets with different SNP densities were constructed (see Table S1). The high-density dataset included populations with available SNP data from Affymetrix arrays; namely African, European, and Mexican HapMap samples [8], Europeans from POPRES [9], West Africans from Bryc et al. [10], and Native Americans from Mao et al. [35]. After merging and quality control filtering, 389,225 SNPs remained and representative population subsets were used in different analyses as detailed through sections below. Our lower density dataset (30,860 SNPs) resulted from the intersection of our high-density dataset with available SNP data generated on Illumina platform arrays, including 52 additional Native American populations [11], as well as additional Latino populations sampled in New York City [7] and 1000 Genomes Latino samples [6]. The resulting dataset combines genomic data for 1,262 individuals from 80 populations. Full details on the population samples are available in Table S1.

### Population structure

An unsupervised clustering algorithm, ADMIXTURE [12], was run on our high-density dataset to explore global patterns of population structure among a representative subset of 641 samples, including seven Native American, eleven POPRES European, HapMap3 Nigerian Yoruba, HapMap3 Mexican, and our six new Caribbean Latino populations (see Table S1). Fourteen ancestral clusters (*K* = 2 through 15) were successively tested. Log likelihoods and cross-validation errors for each *K* clusters are available in Figure S3. F_ST_ based on allele frequencies was calculated in ADMIXTURE v1.22 for each identified cluster at *K* = 8 and values are available in Table S3. Our low-density dataset comprising 1,262 samples (detailed in Table S1) was used to run *K* = 2 through 20. Log likelihoods, cross validation errors and F_ST_ values from ADMIXTURE are available in Figure S9 and Table S4. Principal component analysis (PCA) was applied to both datasets using EIGENSOFT 4.2 [36] and plots were generated using R 2.15.1. Sex bias in ancestry contributions was evaluated by selecting only females (to ensure we compare a diploid X chromosome to diploid autosomes), and running ADMIXTURE at *K* = 3 on the X chromosome and autosomes separately. The Wilcoxon signed rank test, a non-parametric version of the paired Student's t-test that does not require the normality assumption, was applied to assess the significance of the difference in X and autosomal ancestry proportions. This tests whether the average difference of ancestry proportions assigned to a given source population for the X and for the autosomes of each sample is significantly different from zero. The test was applied to the entire collection of Latino samples, revealing an over-arching trend, and then to each population in turn to identify any between-population differences. A rejection of the null hypothesis means that the ancestry proportions on the X and the autosomes are significantly different from one another but does not imply which proportion is larger. We provide box plots as a visual aid to show the direction of the difference (Figure S4). Global ancestry estimates from ADMIXTURE at *K* = 3 were used to test the correlation between male and female ancestry proportions considering all trio founders within each Caribbean population as well as within the full set of admixed trios. Linear models and permutations (up to 100,000) were performed using R 2.15.1.

### Phasing and local ancestry assignment

Family trio genotypes from our six Caribbean populations and continental reference samples were phased using BEAGLE 3.0 software [37]. Local ancestry assignment was performed using PCAdmix (http://sites. google.com/site/pcadmix/ [19]) at *K* = 3 ancestral groups. This approach relies on phased data from reference panels and the admixed individuals. To maintain SNP density and maximize phasing accuracy we restricted to a subset of reference samples with available Affymetrix 6.0 trio data, namely 10 YRI, 10 CEU HapMap3 trios, and 10 Native American trios from Mexico [5]. Each chromosome is analyzed independently, and local ancestry assignment is based on loadings from Principal Components Analysis of the three putative ancestral population panels. The scores from the first two PCs were calculated in windows of 70 SNPs for each panel individual (in previous work we have estimated a suitable number of 10,000 windows to break the genome into when inferring local ancestry using PCAdmix, and in this case, after merging Affymetrix 6.0 data from admixed and reference panels, a total of 743,735 SNPs remained/10,000 = window length of ∼70 SNPs). For each window, the distribution of individual scores within a population is modeled by fitting a multivariate normal distribution. Given an admixed chromosome, these distributions are used to compute likelihoods of belonging to each panel. These scores are then analyzed in a Hidden Markov Model with transition probabilities as in Bryc et al. [10]. The g (generations) parameter in the HMM transition model was determined iteratively so as to maximize the total likelihood of each analyzed population. Local ancestry assignments were determined using a 0.9 posterior probability threshold for each window using the forward-background algorithm. In analyses that required estimating the length of continuous ancestry tracts, the Viterbi algorithm was used. An assessment of the accuracy of this approach is given in [5].

### Tract length analysis

We used the software *Tracts* [20] to identify the migratory model that best explains the genome-wide distribution of ancestry patterns. Specifically, we considered three migration models, each featuring a panmictic population absorbing migrants from three source populations. The models differ by the number of allowed migration events per population. In the simplest model, the population is founded by Native American and European individuals, and later receives a pulse of African migrants. The initial ancestry proportion and timing, as well as the African migration amplitude and timing, are fitted to the data as described below. The other two models feature an additional input of either European or African migrants; the timing and magnitude of this additional pulse result in two additional parameters that must be fitted to the data. Here, the data consisted of Viterbi calls from PCAdmix (see previous section and Figure 2), that is, the most probable assignment of local ancestry along the genomes. To fit parameters to these data, we tallied the inferred continuous ancestry tracts according to inferred ancestry and tract length using 50 equally spaced length bins per population, and one additional bin to account for full chromosomes. Given a migration model and parameters, *Tracts* calculates the expected counts per bin. Assuming that counts in each bin are Poisson distributed, it produces a likelihood estimate that is used to fit model parameters. For each population, we report the model with the best Bayesian Information Criterion (BIC) −2 Log(L)+k Log (n), with n = 153. Because we imposed a fixed number of migration pulses, we must keep in mind that migrations are likely to have been more continuous than what is displayed in the best-fitting models. One way to interpret the pulses are time points that the migrations probably spanned. Resolving the duration of each pulse would likely require refined models and a great deal more data.

### Ancestry-Specific Principal Component Analysis (ASPCA)

To explore within-continent population structure, we applied the following approach for each of the continental ancestries (i.e., Native American, European, and African) of admixed genomes. The general framework is shown in Figure 2. It comprises locus-specific continental ancestry estimation along the genome, followed by PCA analysis restricted to ancestry-specific portions of the genome combined with sub-continental reference panels of ancestral populations. For this purpose, we used our continental-level local ancestry estimates provided by PCAdmix to partition each genome into ancestral haplotype segments, and retained for subsequent analyses only those haplotypes assigned to the continental ancestry of interest. This is achieved by masking (i.e., setting to missing) all segments from the other two continental ancestries. Because ancestry-specific segments may cover different loci from one individual to another, a large amount of missing data results from scaling this approach to a population level, which limits the resolution of PCA. To overcome this problem, we adapted the subspace PCA (ssPCA) algorithm introduced by Raiko et al. [38] to implement a novel ancestry-specific PCA (ASPCA) that allows accommodating phased haploid genomes with large amounts of missing data. Our method is analogous to the ssPCA implementation by Johnson et al. [23], which operates on genotype data. In contrast, ASPCA operates on haplotypes, allowing us to use much more of the genome (rather than just the parts estimated to have two copies of a certain ancestry) and to independently analyze the two haploid genomes of each individual. Finally, ancestry-specific haplotypes derived from admixed individuals are combined with haplotypes derived from putative parental populations and projected together onto PCA space. Details of the ASPCA algorithm and constructed datasets are described in Text S1.

### Differentiation of sub-European ancestry components

To measure the observed deviation in ASPCA of European haplotypes derived from admixed Caribbean populations with respect to the cluster of Iberian samples, a bootstrap resampling-based test was performed. The null distribution was generated from comparing bootstraps of Portuguese and Spanish ASPCA values as models of the intrinsic Iberian population structure. We then compared the ASPCA values of the admixed individuals and tested if the observed differences between Iberian ASPCA values and those of the admixed individuals are more extreme than the differences within Iberia. The distance was determined using the chi-squared statistic of Fisher's method combining ASPC1 and ASPC2 t-tests for each bootstrap. We ran 10,000 bootstraps to determine one-tailed *p*-values. As Iberians we considered: POPRES Spanish, POPRES Portuguese, Andalusians, and Galicians; and as Caribbean Latinos: CUB, PUR, DOM, COL, and HON. Additional tests were performed comparing Portuguese versus the rest of Iberians and between an independent dataset of Mexican individuals analyzed by Moreno-Estrada, Gignoux et al. (in preparation) projected onto ASPCA space using the same reference panel of European populations. A bivariate test was performed to measure the relative deviation from the Iberian cluster of the distribution given by the Caribbean versus the Mexican dataset. To determine whether insular versus mainland Caribbean populations disperse over significantly different ranges in ASPC2, a Wilcoxon rank test was performed between (COL+HON) versus (CUB, PUR, DOM). Haitians were excluded due to low sample size (N = 2 haplotypes). Boxplot is available in Figure S11. Population differentiation estimates between clusters inferred with ADMIXTURE were visualized and compared across runs where both the Latino-specific and southern European components were detected. Values are available in Table S3 and Table S4. To provide independent evidence on the sub-continental ancestry of European haplotypes, we considered segments that are identical by descent (IBD) between unrelated Latino individuals and a representative subset of European populations. We used our high-density dataset to extract a subset of 203 POPRES European individuals and the founders of the 65 complete admixed trios. We first performed a genome-wide pairwise IBS estimation using PLINK [39] to ensure that the dataset contains no samples with more than 10% IBS with any other sample. Then we used fastIBD [37] to phase the data and estimate segments shared IBD longer than 2 Mb to eliminate false positive IBD matches and assuming that ancestry will be shared among pairwise IBD hits of segments this long. All 2 Mb or greater segments shared IBD between pairs of individuals were summed, and histograms were created for pairwise matches within each group (i.e., POPRES Europeans, Iberians, and Caribbean Latinos). To inform about the proportion of shared DNA between pairs of populations we calculated a summed pairwise IBD statistic, which is the sum of lengths of all segments inferred to be shared IBD between a given European population and each Latino population, normalized by sample size.

### Size-based ASPCA analyses

Given the evidence from our tract length analysis for a second pulse of African migrants into the admixture of insular Caribbean Latinos, a modified size-based ASPCA analysis was performed. A reference panel was built integrating three different resources [8], [10], [40] and focusing on putative source populations from along the West African coast, including Mandenka from Senegal, Yoruba and Igbo from Nigeria, Bamoun and Fang from Cameroon, Brong from western Ghana, and Kongo from the Democratic Republic of the Congo. We begin with the continental local ancestry inference from PCAdmix *K* = 3. For each individual we then divide African ancestry tracts into small (0 to 50 cM) and large (>50 cM) size classes. Given a partition of African ancestry tracts, we take all sites included in one tract class, say short tracts, and run PCA on our sub-continental West African reference populations for only these sites. Using the first two PCs from this analysis, we fit a bivariate normal distribution to each reference population cluster. We then project our test sample into this PCA space, and estimate the probability of it coming from each reference population using the fitted distributions. This procedure is repeated for each tract class, for each individual. For each admixed Caribbean population, we can then estimate the probability that a given class of African ancestry tracts comes from a specific West African source population as the average probability of assignment to this population across all individuals. Finally, under the assumption that a given class of African tracts must come from one of the provided reference populations, we rescale these probabilities to sum to one. Each assignment estimate is also provided with error bars representing the standard error of the mean. We compare the short and long assignment probabilities for each Caribbean population to identify distinct sources for “older” and “younger” West African migratory source populations. Haitians were not included in the analysis due to low sample size (*n* = 4). Due to concerns that shorter tracts have a higher likelihood of mis-assignment, we added a medium tract size class (5 cM to 50 cM) to see if the results were simply due to very short (0 cM to 5 cM) European or Native American tracts being mis-classified as African. We compare the results for short and medium tracts and find that the trends are maintained suggesting the observation that older shorter tracts appear to be primarily from the Mandenka and Brong source populations is not simply due to short tract mis-assignment

### Local ancestry estimation within African tracts

To identify likely regions of Yoruba versus Mandenka ancestry in the African component, we modified our implementation of PCAdmix to perform local ancestry deconvolution solely of the African segments of the admixed genomes. The modification is achieved in the final step of the algorithm: whereas the standard approach estimates a single HMM across an entire chromosome, here we fit J disjoint HMMs spanning each of the J blocks of African ancestry in a given chromosome for a given individual. Applying the method, we obtained posterior probabilities for Mandenka versus Yoruba ancestry within the previously inferred African segments. We then selected only those sub-regions that were confidently called as Mandenka or Yoruba, and stratified them by physical size.

## Supporting Information

Figure S1 Principal component 1 versus lower order PCs defining sub-continental components among Native American populations. Top: PC5 separates Venezuelan population isolates from the rest of Native Americans. Bottom: PC7 separates Mesoamerican from Andean groups. Mexicans and Hondurans distribute between the European and Mesoamerican clusters, whereas Colombians slightly deviate towards the Andean and Venezuelan clusters. Global PCA analysis based on the high-density dataset (∼390 K SNPs) and thus limited to reference panel populations with available Affymetrix SNP array data (see Table S1 for details).
(TIF)

Figure S2 ADMIXTURE results from *K* = 2 through 15 based on the high-density dataset (∼390 K SNPs) including 7 admixed Latino populations and 19 reference populations. A low-frequency Southern European component restricted to Mediterranean populations at lower order Ks and specifically to Iberian populations at higher order Ks, accounts for the majority of European ancestry among Latinos (black bars). It further decomposes into population-specific clusters (purple bars) denoting higher similarities within the European portion among Latinos compared to European source populations.
(TIF)

Figure S3 ADMIXTURE metrics at increasing K values based on Log-likelihoods (A) and cross-validation errors (B) for results shown in Figure S2.
(TIF)

Figure S4 Comparison of ADMIXTURE estimates obtained from autosomes and the X chromosome in different Latino/Caribbean populations. A) Cluster-based results for *K* = 3 using the same set of ancestral populations as in Figure S2. Because the X chromosome is diploid, the analysis was restricted to female individuals from the seven admixed Latino populations. Within each population, individuals are sorted from largest to smallest proportion of European ancestry. B) Box plot showing the directionality of the difference between X and autosomal ancestry proportions considering all populations together. *P*-values on top correspond to the Wilcoxon signed rank test applied to assess statistical significance (see Materials and Methods). C) Box plots and statistical tests for each population (Haitians excluded due to low sample size). The observed pattern strongly supports the presence of sex-biased gene flow during the process of admixture throughout the Caribbean, with significantly higher contribution from Native American, and to a lesser extent West African, ancestors into the composition of the X chromosome, which largely reflects the female demographic history of a population.
(TIF)

Figure S5 Correlation between male and female continental ancestries. Parents' ancestry proportions from each trio were used to compare correlation coefficients between the observed values and 100,000 permuted male-female pairs (*p*-values shown for the combined set of Latino Caribbean samples and for each population in Table S2).
(TIF)

Figure S6 Ancestry tract lengths distribution per population and demographic model tested in *Tracts*. For each demographic scenario, the observed distribution is compared to the predictions of the best-fitting migration model (displayed below each distribution). Solid lines represent model predictions and shaded areas are one-sigma confidence region surrounding the predictions. Three different demographic scenarios were considered, all of which assume the involvement of European and Native American tracts at the onset of admixture, followed by the introduction of African migrants (denoted by *EUR,NAT+AFR*). The second and third models allow for an additional pulse of European (*EUR,NAT+AFR+EUR*) and African (*EUR,NAT+AFR+AFR*) ancestry, respectively. Likelihood values for each model are shown on top of each plot. Pie charts above each migration model are proportional to the estimated number of migrants being introduced at each point in time (black arrows). GA: generations ago.
(TIF)

Figure S7 ASPCA analysis of Native American haplotypes derived from admixed genomes (solid circles) and reference panel populations from [11] grouped by linguistic families as reported therein. Top panels: ASPCA with the full reference panel of Native American populations. Bottom panels: Filtered ASPCA without extreme outliers (Aleutians, Greenlanders, and Surui excluded from the analysis). Each individual from the reference panel is represented by the corresponding population label centered on its PCA coordinates. A zoomed version of PC1 vs. PC2 for the filtered set (bottom left) grouped by geographic sampling location is available in Figure 4A.
(TIF)

Figure S8 ADMIXTURE results from *K* = 2 through 20 based on the low-density dataset (∼30 K SNPs) including additional admixed Latino and Native American reference populations (see Table S1 for details). The presence of the Latino European component (black and gray bars) is recaptured among independently sampled Latino populations. FL: Florida (this study); NY: New York; 1KG: 1000 Genomes Project samples. Native American populations from [11] are grouped according to linguistic families reported therein. Labels are shown for the populations representing the 15 Native American clusters identified at *K* = 20 (four of the remaining five being of European ancestry and one of West African ancestry). Clusters involving multiple populations are identified by those with the highest membership values. Throughout lower and higher order Ks, several South American components (yellow and green bars), show varying degrees of shared genetic membership with Mesoamerican Mayans, accounting for up to nearly half of their genome composition (see Figure 4 for more details).
(TIF)

Figure S9 ADMIXTURE metrics at increasing K values based on Log-likelihoods (A) and cross-validation errors (B) for results shown in Figure S8.
(TIF)

Figure S10 ASPCA distribution of Iberian samples (red circles) compared to European haplotypes derived from our Latino Caribbean samples (top panel) and from an independent cohort of Mexican samples (bottom panel). The relative deviation from the Iberian cluster is significantly different comparing the Caribbean versus the Mexican dataset (see the main text for details).
(TIF)

Figure S11 ASPC2 values per population from the European-specific PCA analysis shown in Figure 5 and Figure S10. Population codes as in Table S1. The boxplot shows that low ASPC2 values are enriched with mainland Colombian and Honduran haplotypes, whereas insular Caribbean populations show less deviated values from the Iberian cluster. A Wilcoxon rank test between mainland (COL, HON) versus insular samples (CUB, PUR, DOM) demonstrated that these two groups disperse over significantly different ranges in ASPC2 (Haitians excluded due to low sample size).
(TIF)

Figure S12 IBD sharing between different Caribbean Latino populations and a representative subset of POPRES European populations as measured by a summed pairwise IBD statistic. For each Latino population, maximum pairwise IBD levels were observed in those pairs involving Spanish and, to a lesser extent, Portuguese samples, in agreement with our ASPCA results.
(TIF)

Figure S13 IBD sharing between pairs of individuals within A) Caribbean Latinos and B) a representative subset of POPRES European populations. Inset histograms display counts lower than 50 for the same binning categories. The overall count of pairs sharing short segments of total IBD is higher among Europeans, probably as a result of an older shared pool of source haplotypes. In contrast, the higher frequency of longer IBD matches among Latinos is compatible with a recent European founder effect. After excluding within-population pairs of Latino individuals (top right), there are still more and longer IBD matches among Caribbean populations compared to Iberians (bottom right).
(TIF)

Figure S14 ASPCA analysis of African haplotypes derived from admixed genomes with >25% of African ancestry (black symbols) and a representative subset of African HapMap3 and other West African reference panel populations from [10]. Colombians and Hondurans excluded due to lower overall proportions of African ancestry.
(TIF)

Figure S15 ASPCA analysis of short versus long African ancestry tracts from admixed genomes and West African reference panel populations. To exemplify our size-based ASPCA approach, the African genome of a Puerto Rican individual is displayed (denoted by PUR). Left: PUR clusters with Mandenka when only sites within short ancestry tracts (<50 cM) are considered to perform PCA. Right: a similar background distribution is obtained but the same PUR individual no longer clusters with Mandenka when considering long ancestry tracts (>50 cM).
(TIF)

Figure S16 African ancestry size-based ASPCA results per population sample. Considering three different classes of ancestry tract lengths (black: short; red: long; blue: intermediate), scaled assignment probabilities are shown for each African source population. Values on the y-axis are the average probability of assignment to each potential source population across all individuals within each Latino population (see Materials and Methods for details).
(TIF)

Table S1 Summary of Latino populations and assembled reference panels.
(PDF)

Table S2 Correlation *p*-values of male vs. female ancestry.
(PDF)

Table S3 F_ST_ divergences between estimated populations for *K* = 8 using ADMIXTURE.
(PDF)

Table S4 F_ST_ divergences between estimated populations for *K* = 20 using ADMIXTURE.
(PDF)

Text S1 Methodology of the Ancestry-Specific PCA (ASPCA) implementation.
(PDF)
