# Supplementary figures and images for: Reconstructing the Population Genetic History of the Caribbean
Source: PLoS Genet. 2013 Nov 14;9(11):e1003925. doi: 10.1371/journal.pgen.1003925 (PMC3828151; doi:10.1371/journal.pgen.1003925)

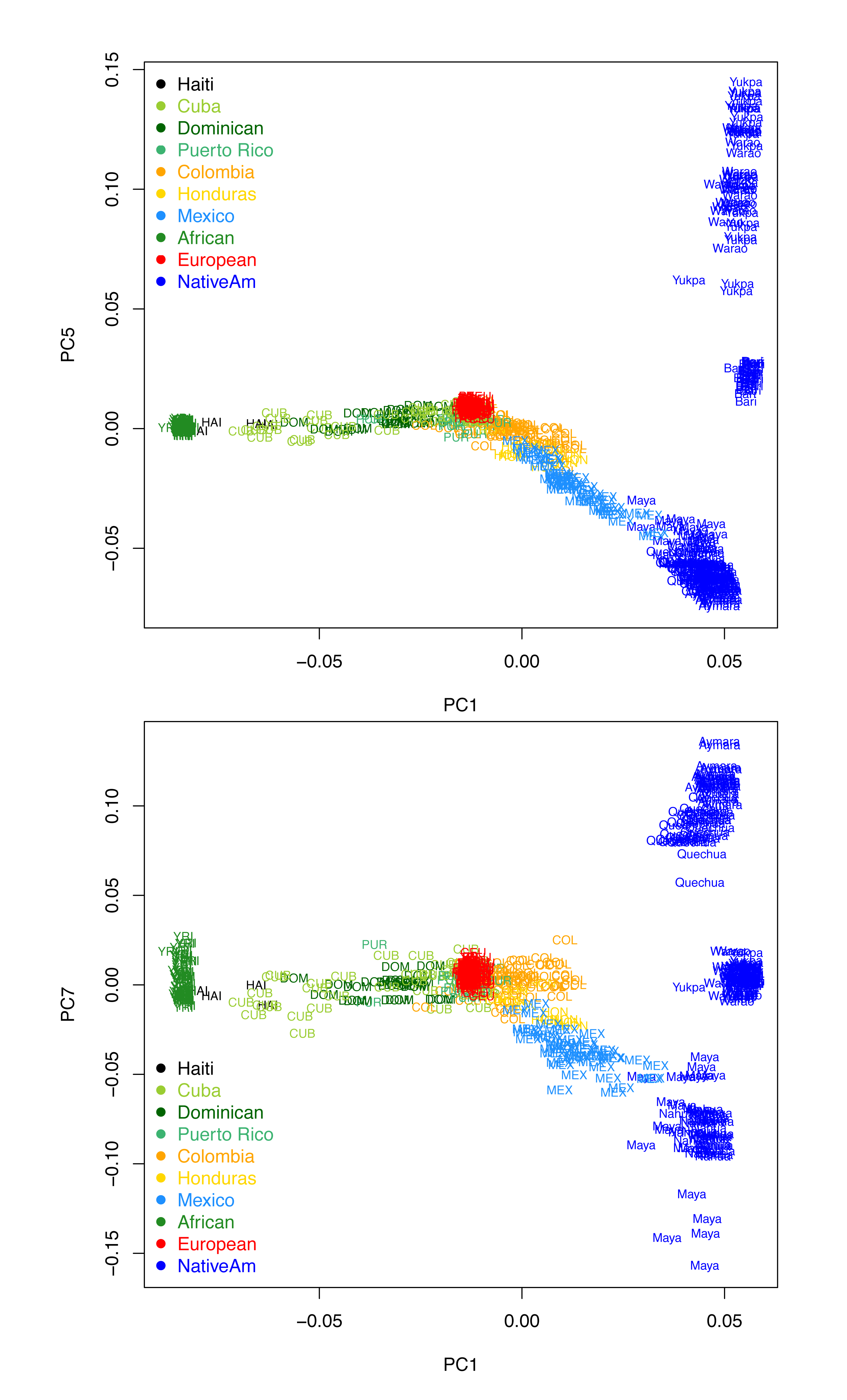

Supplement: Figure S1 — Principal component 1 versus lower order PCs defining sub-continental components among Native American populations. Top: PC5 separates Venezuelan population isolates from the rest of Native Americans. Bottom: PC7 separates Mesoamerican from Andean groups. Mexicans and Hondurans distribute between the European and Mesoamerican clusters, whereas Colombians slightly deviate towards the Andean and Venezuelan clusters. Global PCA analysis based on the high-density dataset (∼390 K SNPs) and thus limited to reference panel populations with available Affymetrix SNP array data (see Table S1 for details). (TIF) [file pgen.1003925.s001.tif]

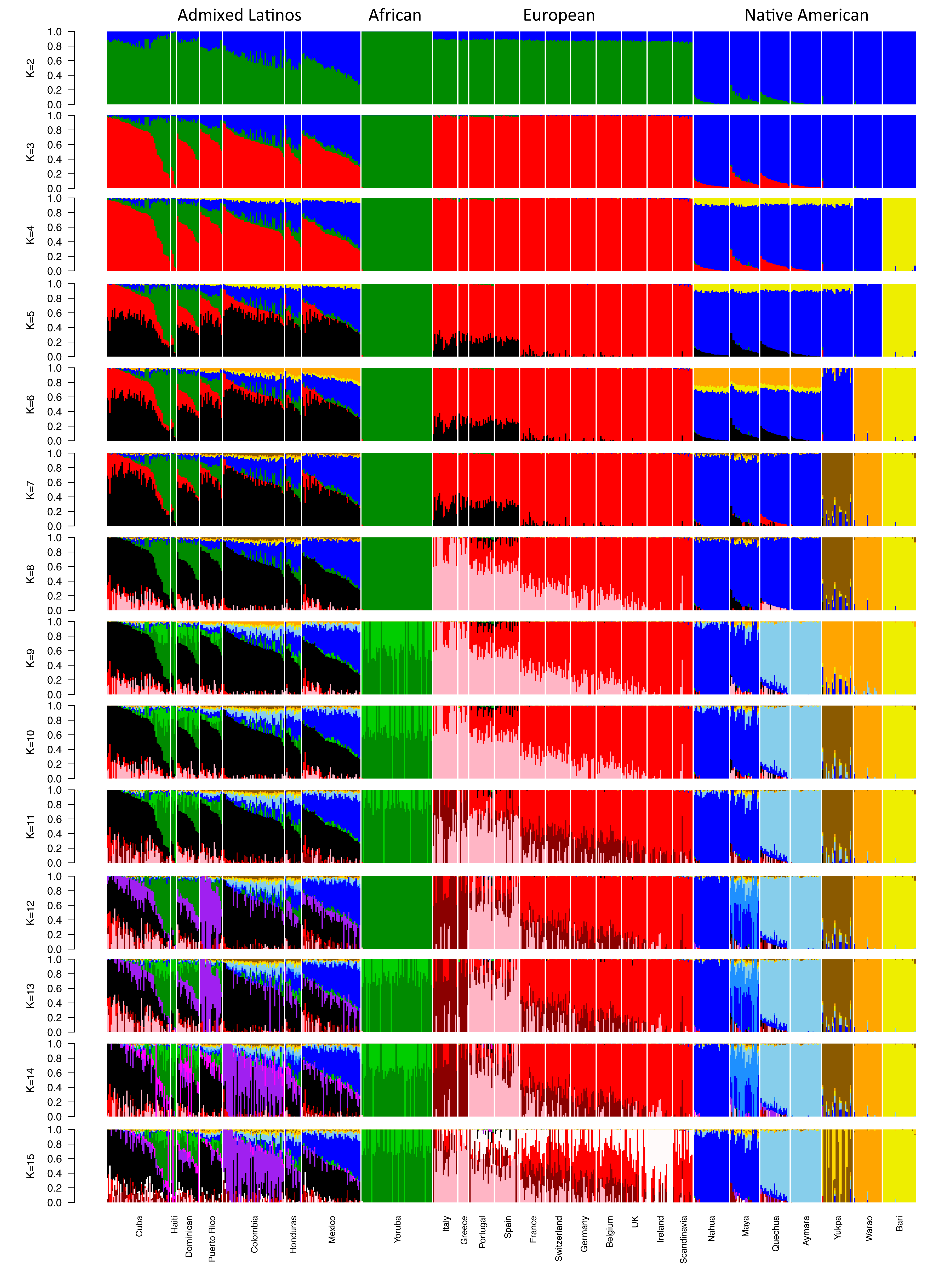

Supplement: Figure S2 — ADMIXTURE results from K = 2 through 15 based on the high-density dataset (∼390 K SNPs) including 7 admixed Latino populations and 19 reference populations. A low-frequency Southern European component restricted to Mediterranean populations at lower order Ks and specifically to Iberian populations at higher order Ks, accounts for the majority of European ancestry among Latinos (black bars). It further decomposes into population-specific clusters (purple bars) denoting higher similarities within the European portion among Latinos compared to European source populations. (TIF) [file pgen.1003925.s002.tif]

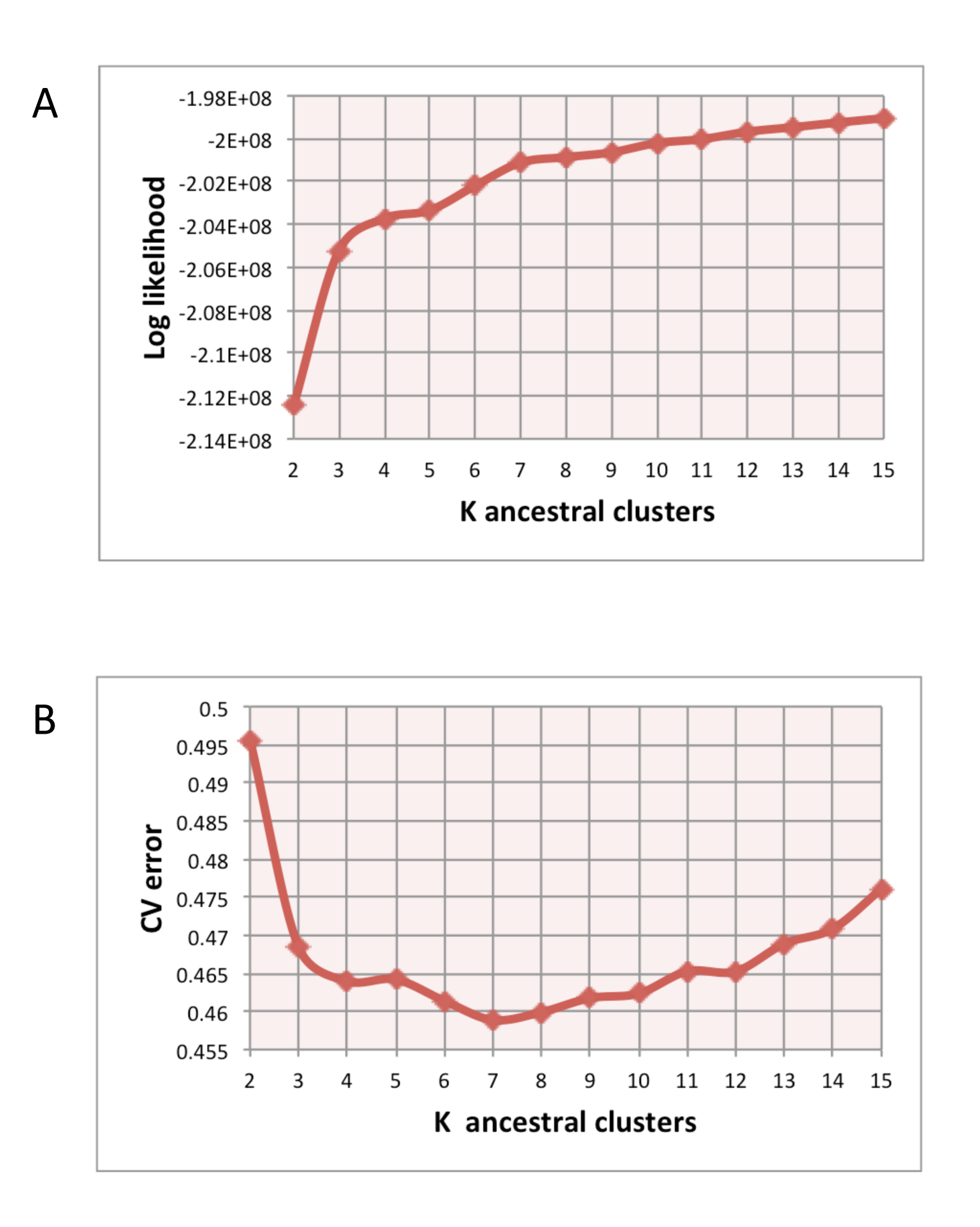

Supplement: Figure S3 — ADMIXTURE metrics at increasing K values based on Log-likelihoods (A) and cross-validation errors (B) for results shown in Figure S2. (TIF) [file pgen.1003925.s003.tif]

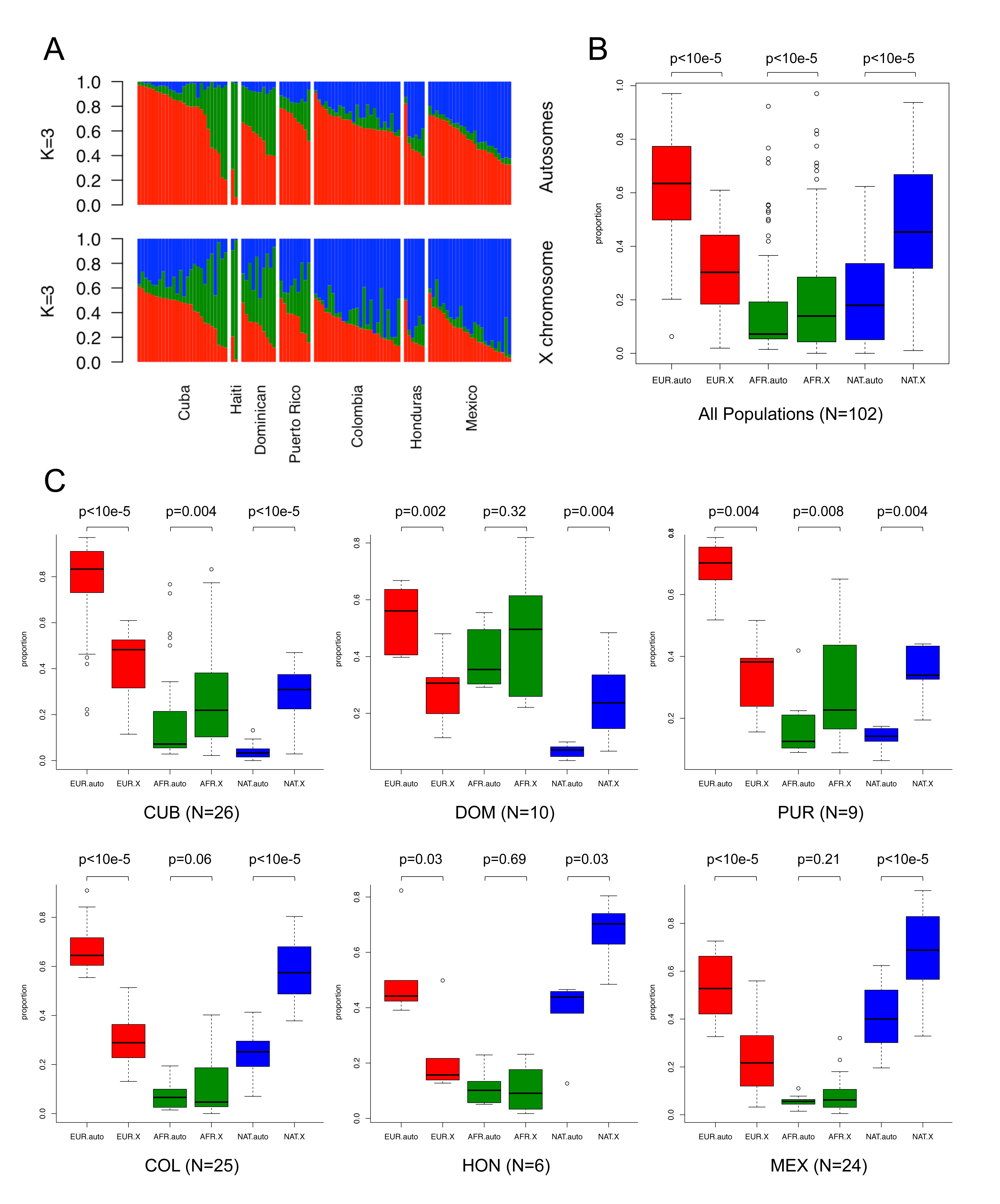

Supplement: Figure S4 — Comparison of ADMIXTURE estimates obtained from autosomes and the X chromosome in different Latino/Caribbean populations. A) Cluster-based results for K = 3 using the same set of ancestral populations as in Figure S2. Because the X chromosome is diploid, the analysis was restricted to female individuals from the seven admixed Latino populations. Within each population, individuals are sorted from largest to smallest proportion of European ancestry. B) Box plot showing the directionality of the difference between X and autosomal ancestry proportions considering all populations together. P-values on top correspond to the Wilcoxon signed rank test applied to assess statistical significance (see Materials and Methods). C) Box plots and statistical tests for each population (Haitians excluded due to low sample size). The observed pattern strongly supports the presence of sex-biased gene flow during the process of admixture throughout the Caribbean, with significantly higher contribution from Native American, and to a lesser extent West African, ancestors into the composition of the X chromosome, which largely reflects the female demographic history of a population. (TIF) [file pgen.1003925.s004.tif]

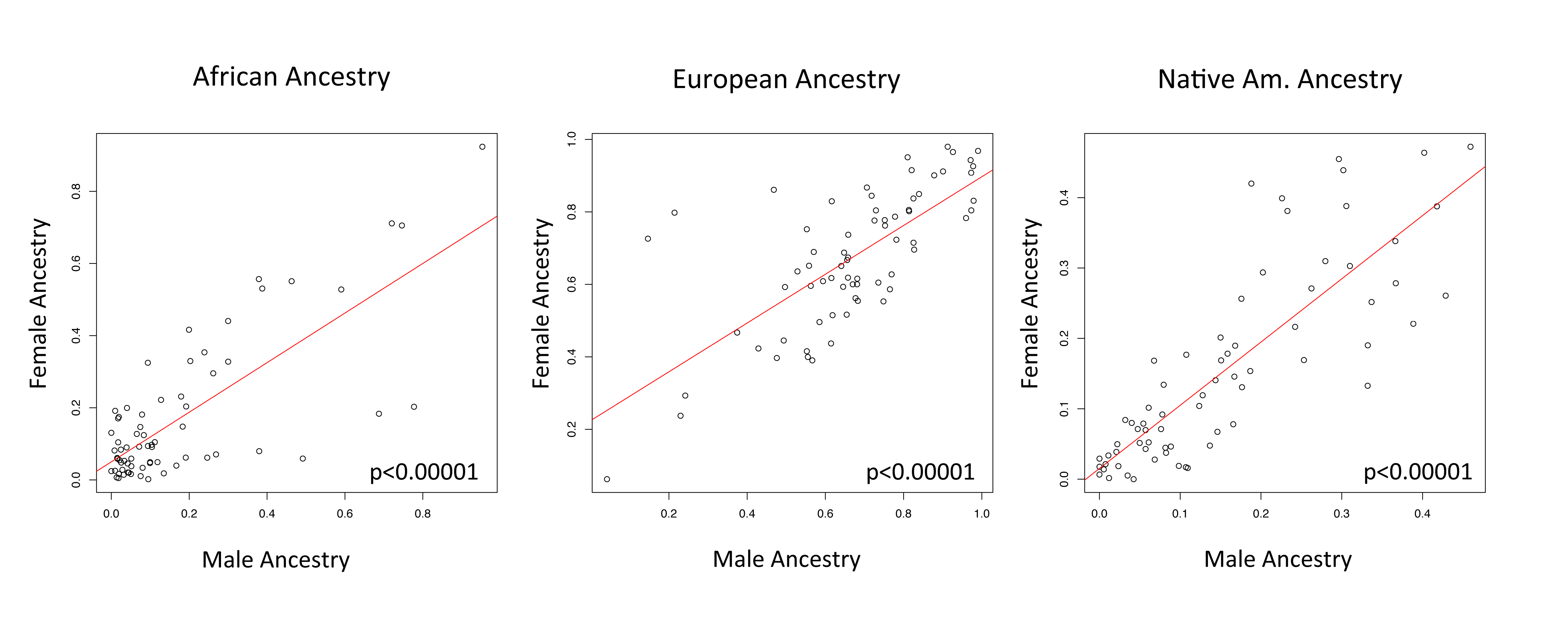

Supplement: Figure S5 — Correlation between male and female continental ancestries. Parents' ancestry proportions from each trio were used to compare correlation coefficients between the observed values and 100,000 permuted male-female pairs (p-values shown for the combined set of Latino Caribbean samples and for each population in Table S2). (TIF) [file pgen.1003925.s005.tif]

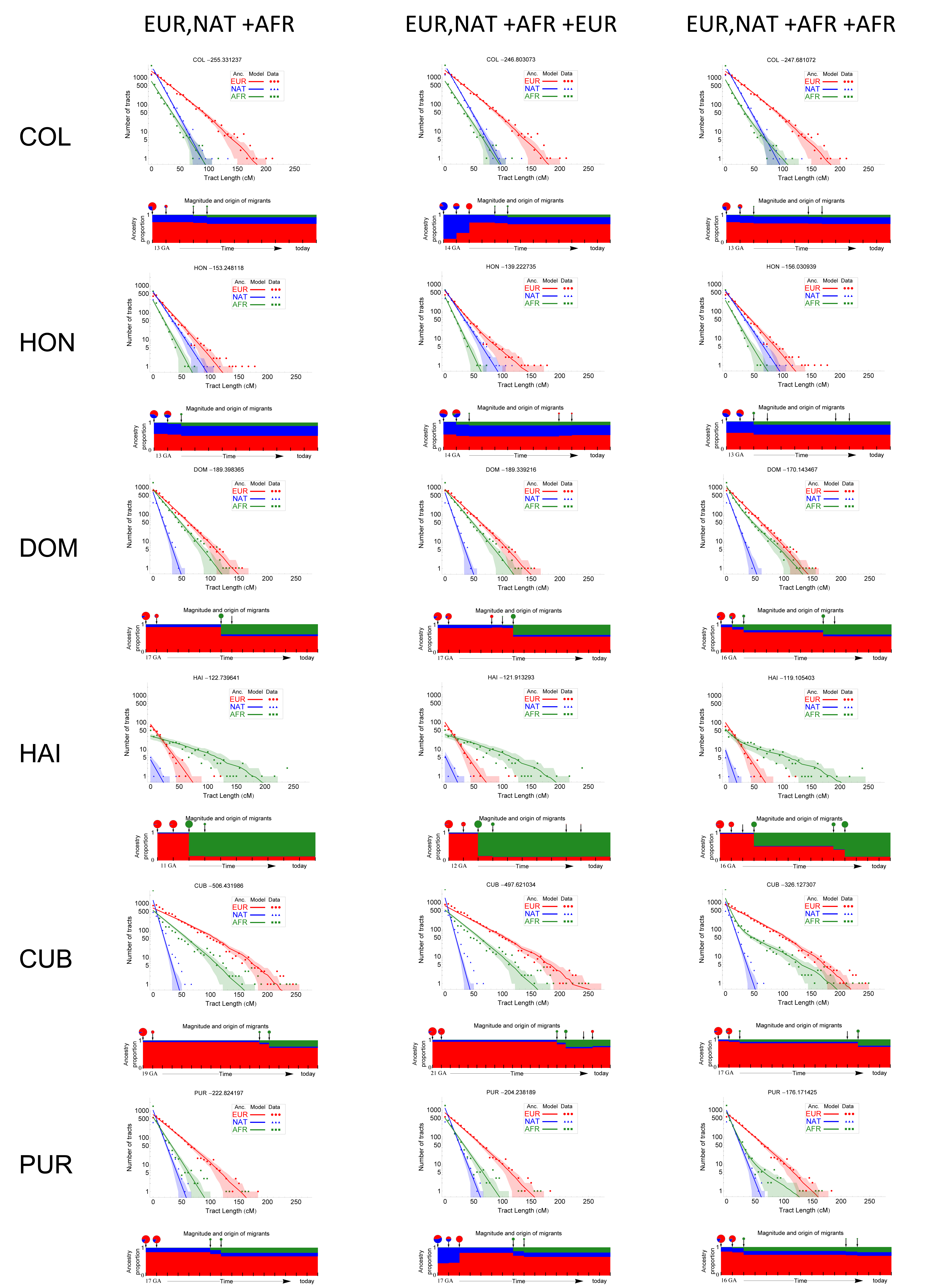

Supplement: Figure S6 — Ancestry tract lengths distribution per population and demographic model tested in Tracts. For each demographic scenario, the observed distribution is compared to the predictions of the best-fitting migration model (displayed below each distribution). Solid lines represent model predictions and shaded areas are one-sigma confidence region surrounding the predictions. Three different demographic scenarios were considered, all of which assume the involvement of European and Native American tracts at the onset of admixture, followed by the introduction of African migrants (denoted by EUR,NAT+AFR). The second and third models allow for an additional pulse of European (EUR,NAT+AFR+EUR) and African (EUR,NAT+AFR+AFR) ancestry, respectively. Likelihood values for each model are shown on top of each plot. Pie charts above each migration model are proportional to the estimated number of migrants being introduced at each point in time (black arrows). GA: generations ago. (TIF) [file pgen.1003925.s006.tif]

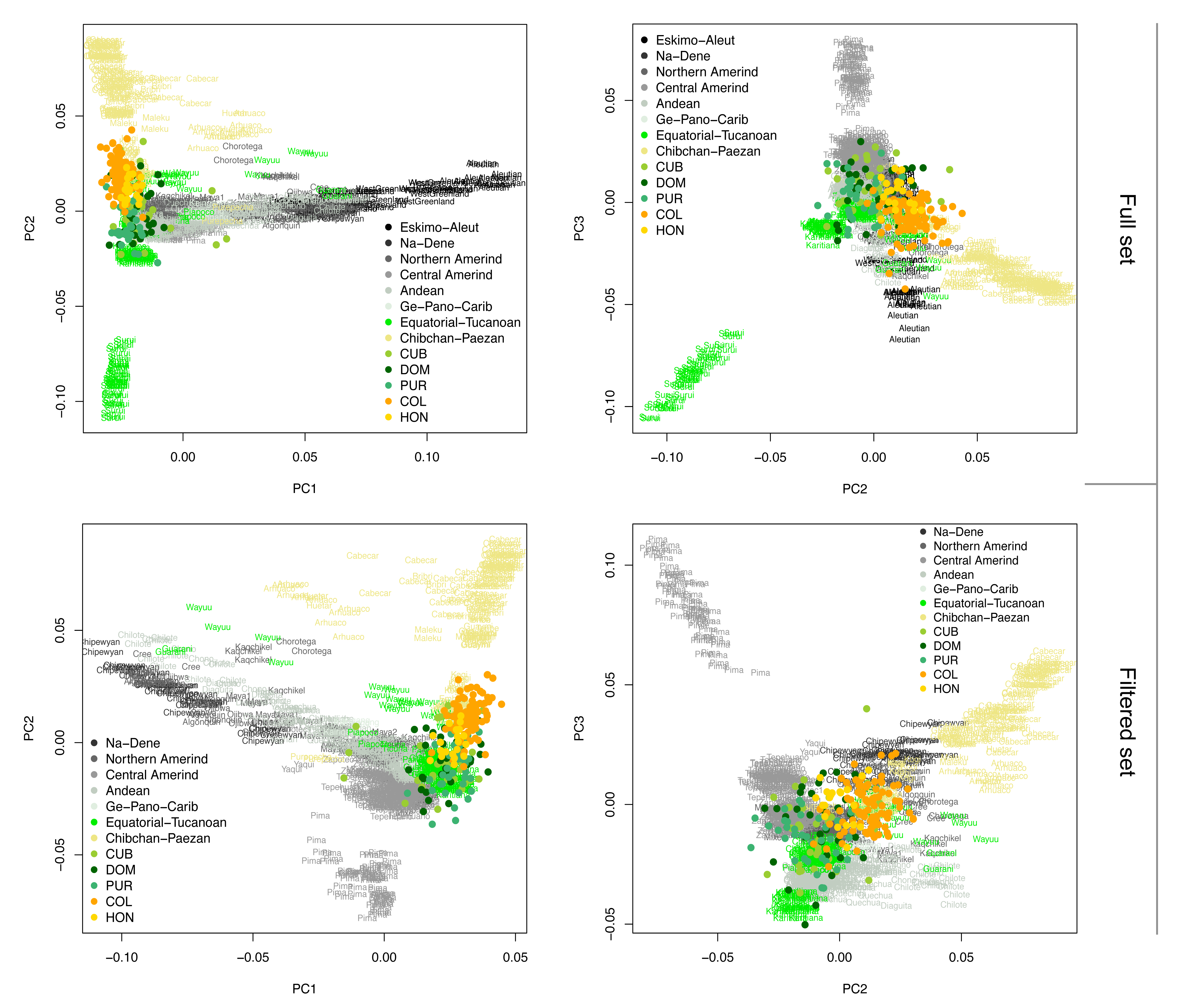

Supplement: Figure S7 — ASPCA analysis of Native American haplotypes derived from admixed genomes (solid circles) and reference panel populations from [11] grouped by linguistic families as reported therein. Top panels: ASPCA with the full reference panel of Native American populations. Bottom panels: Filtered ASPCA without extreme outliers (Aleutians, Greenlanders, and Surui excluded from the analysis). Each individual from the reference panel is represented by the corresponding population label centered on its PCA coordinates. A zoomed version of PC1 vs. PC2 for the filtered set (bottom left) grouped by geographic sampling location is available in Figure 4A. (TIF) [file pgen.1003925.s007.tif]

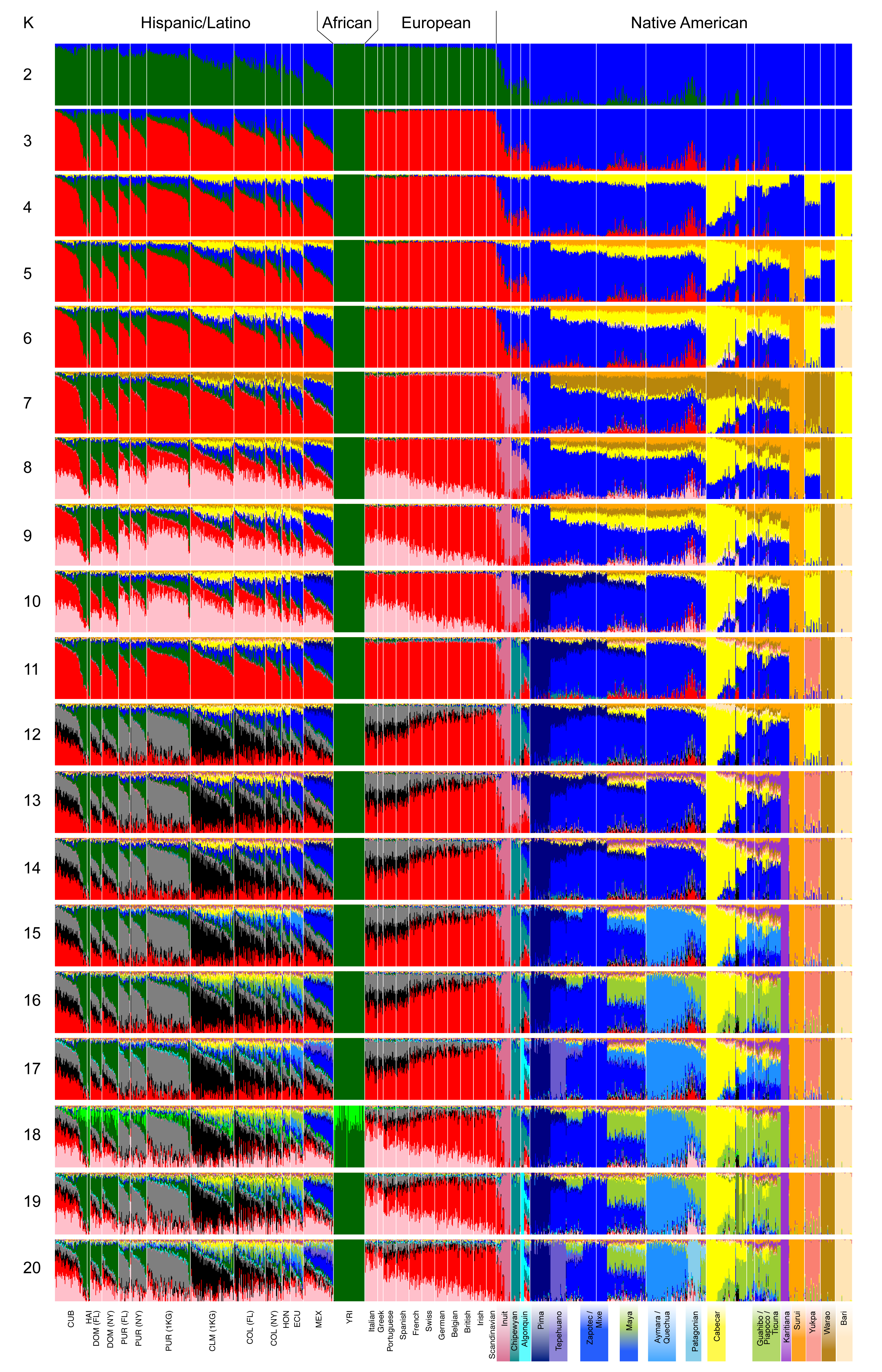

Supplement: Figure S8 — ADMIXTURE results from K = 2 through 20 based on the low-density dataset (∼30 K SNPs) including additional admixed Latino and Native American reference populations (see Table S1 for details). The presence of the Latino European component (black and gray bars) is recaptured among independently sampled Latino populations. FL: Florida (this study); NY: New York; 1KG: 1000 Genomes Project samples. Native American populations from [11] are grouped according to linguistic families reported therein. Labels are shown for the populations representing the 15 Native American clusters identified at K = 20 (four of the remaining five being of European ancestry and one of West African ancestry). Clusters involving multiple populations are identified by those with the highest membership values. Throughout lower and higher order Ks, several South American components (yellow and green bars), show varying degrees of shared genetic membership with Mesoamerican Mayans, accounting for up to nearly half of their genome composition (see Figure 4 for more details). (TIF) [file pgen.1003925.s008.tif]

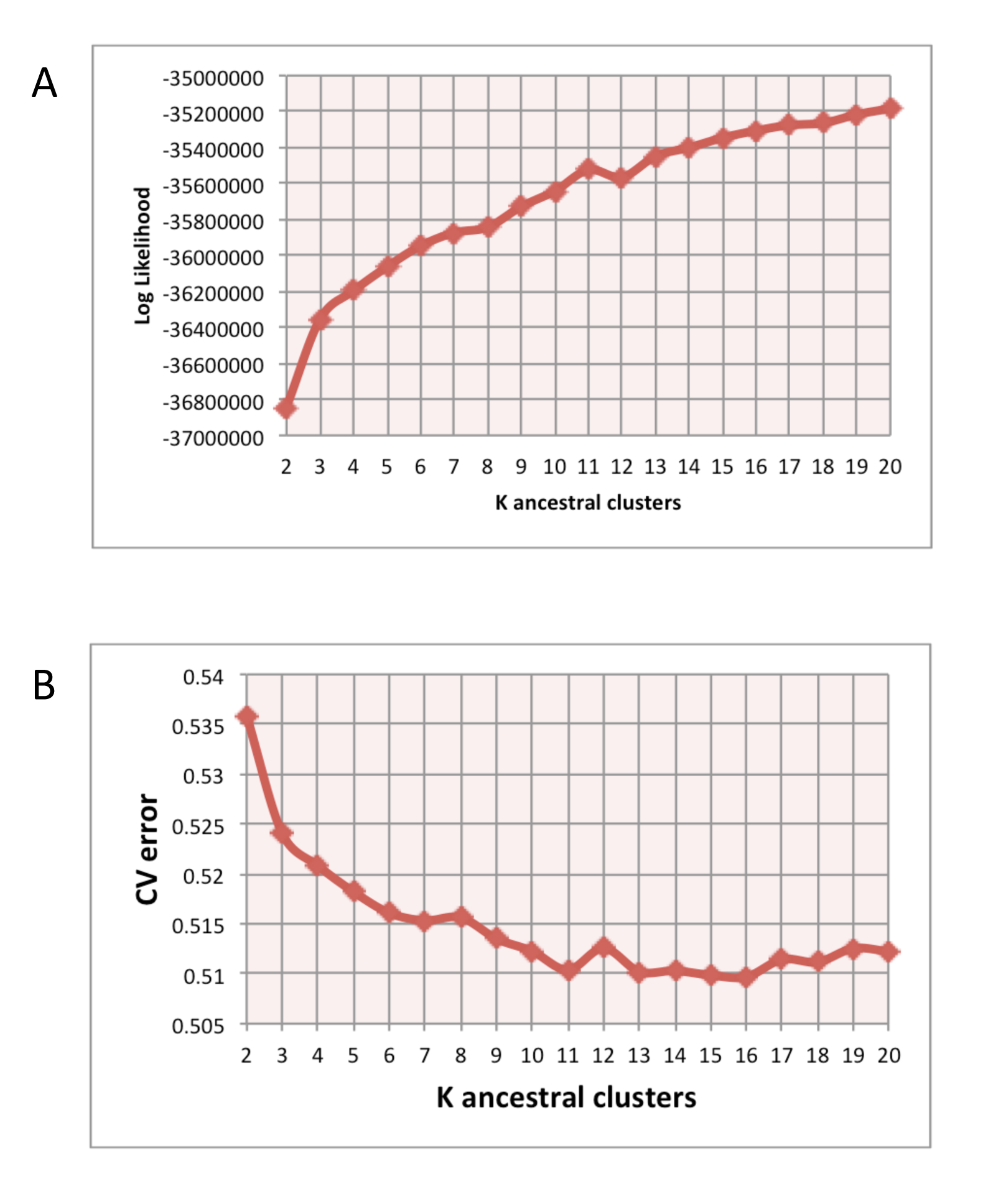

Supplement: Figure S9 — ADMIXTURE metrics at increasing K values based on Log-likelihoods (A) and cross-validation errors (B) for results shown in Figure S8. (TIF) [file pgen.1003925.s009.tif]

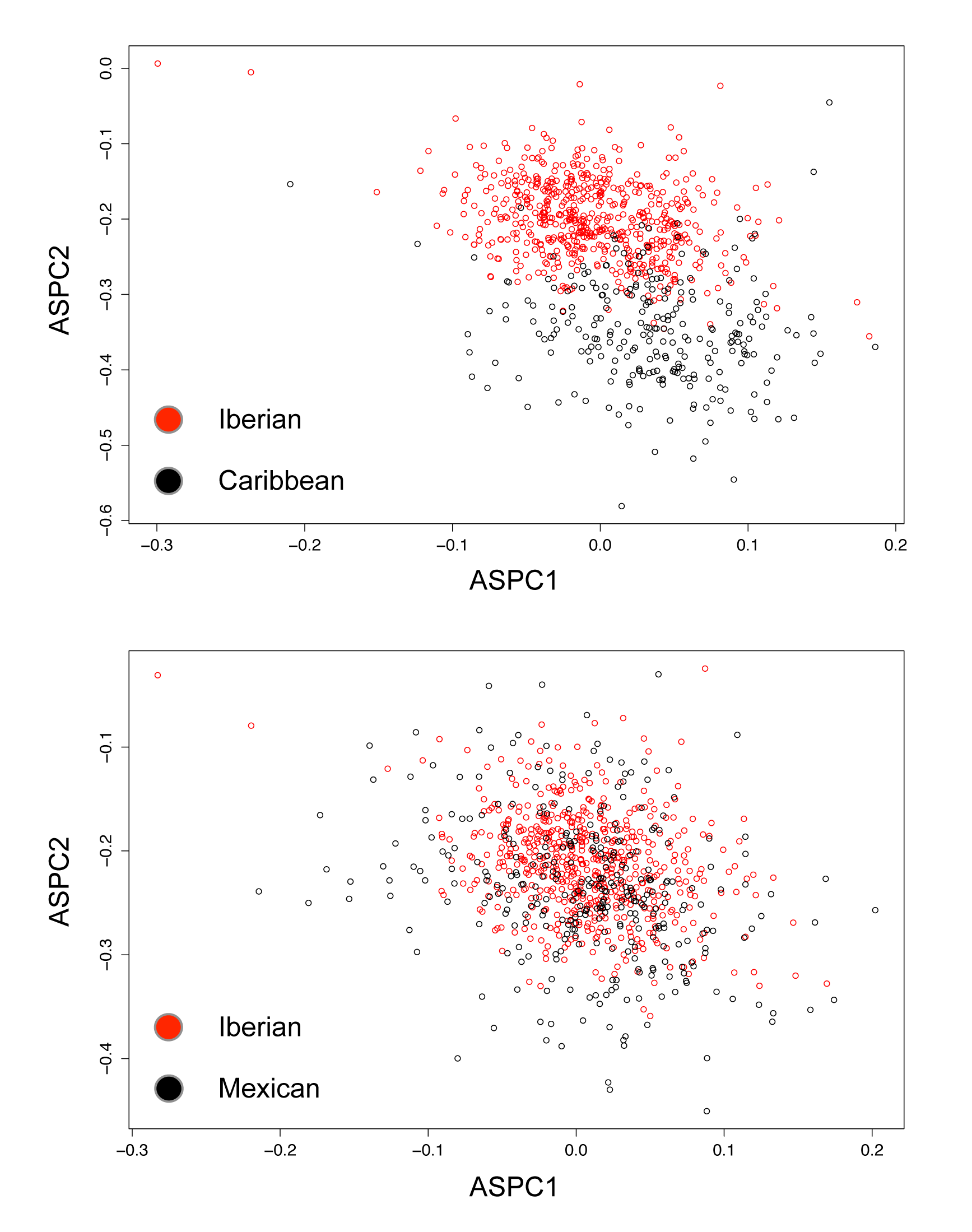

Supplement: Figure S10 — ASPCA distribution of Iberian samples (red circles) compared to European haplotypes derived from our Latino Caribbean samples (top panel) and from an independent cohort of Mexican samples (bottom panel). The relative deviation from the Iberian cluster is significantly different comparing the Caribbean versus the Mexican dataset (see the main text for details). (TIF) [file pgen.1003925.s010.tif]

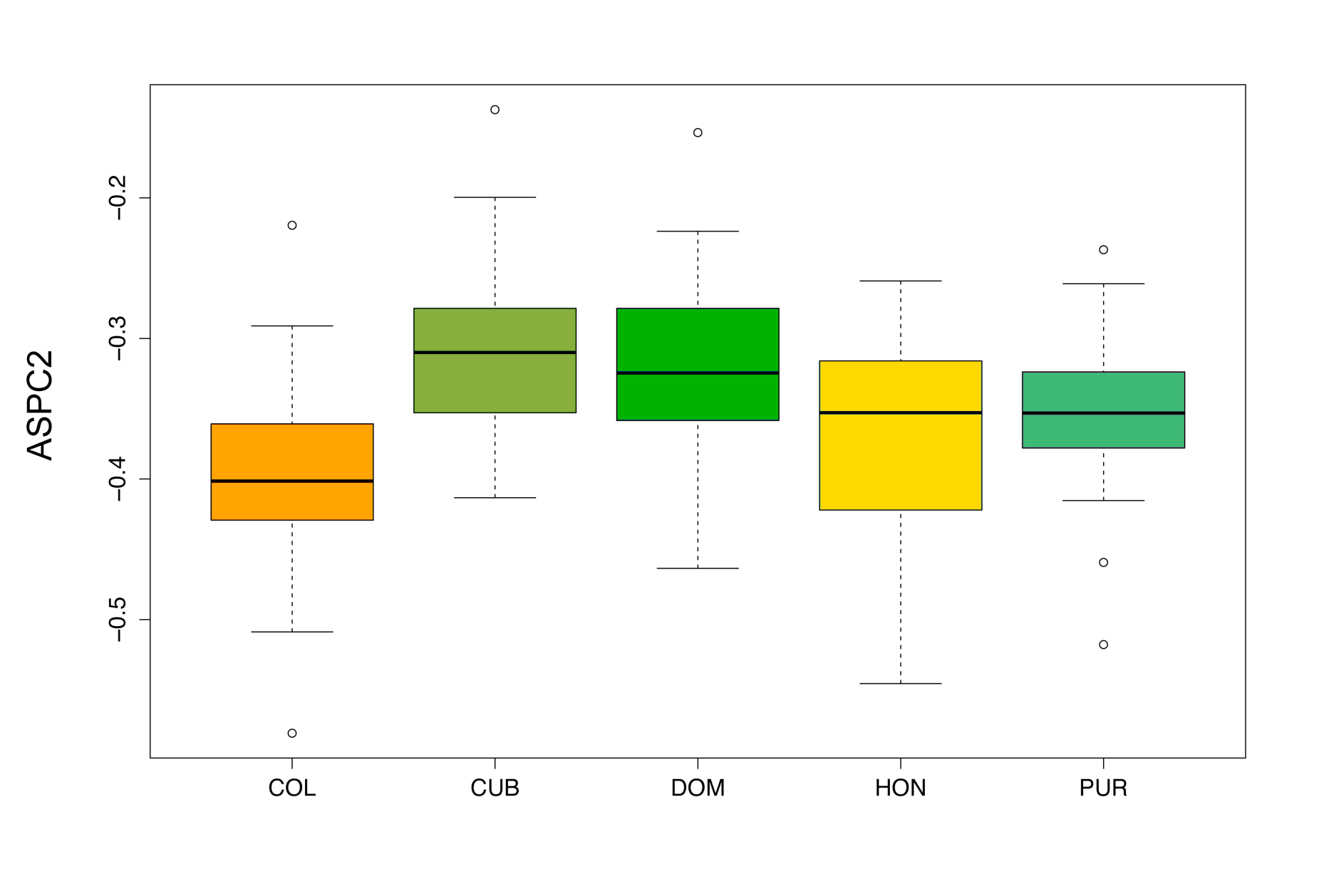

Supplement: Figure S11 — ASPC2 values per population from the European-specific PCA analysis shown in Figure 5 and Figure S10. Population codes as in Table S1. The boxplot shows that low ASPC2 values are enriched with mainland Colombian and Honduran haplotypes, whereas insular Caribbean populations show less deviated values from the Iberian cluster. A Wilcoxon rank test between mainland (COL, HON) versus insular samples (CUB, PUR, DOM) demonstrated that these two groups disperse over significantly different ranges in ASPC2 (Haitians excluded due to low sample size). (TIF) [file pgen.1003925.s011.tif]

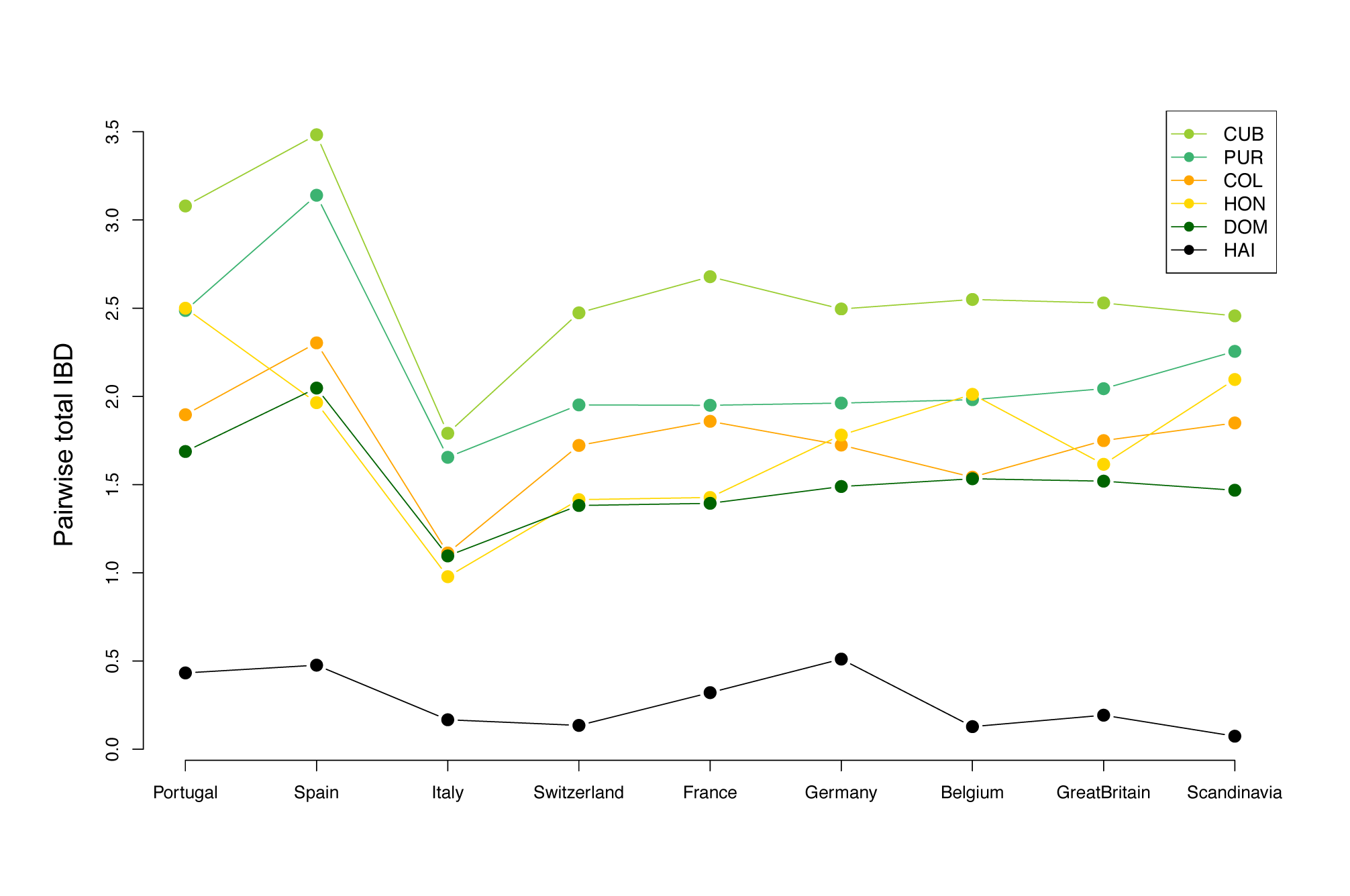

Supplement: Figure S12 — IBD sharing between different Caribbean Latino populations and a representative subset of POPRES European populations as measured by a summed pairwise IBD statistic. For each Latino population, maximum pairwise IBD levels were observed in those pairs involving Spanish and, to a lesser extent, Portuguese samples, in agreement with our ASPCA results. (TIF) [file pgen.1003925.s012.tif]

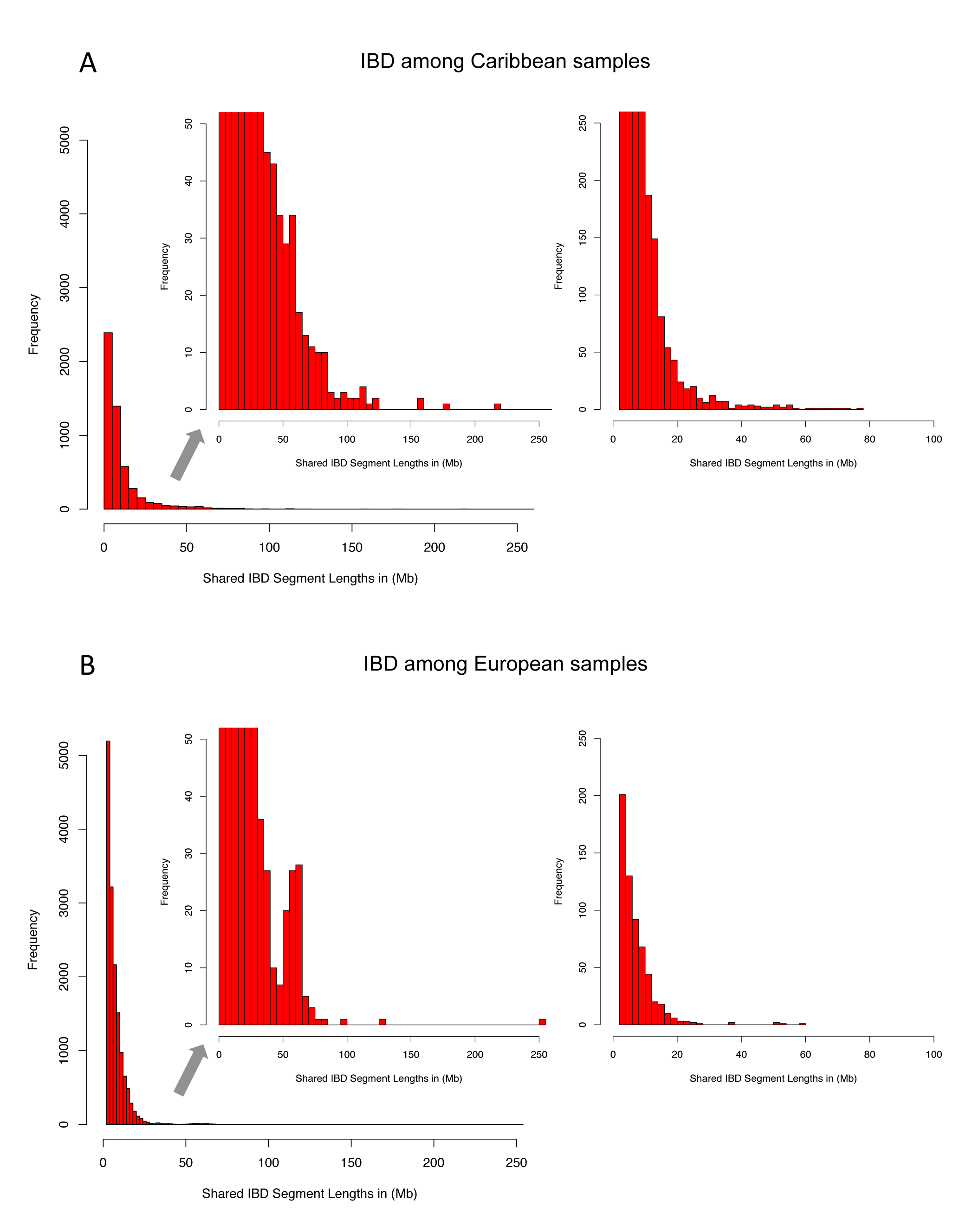

Supplement: Figure S13 — IBD sharing between pairs of individuals within A) Caribbean Latinos and B) a representative subset of POPRES European populations. Inset histograms display counts lower than 50 for the same binning categories. The overall count of pairs sharing short segments of total IBD is higher among Europeans, probably as a result of an older shared pool of source haplotypes. In contrast, the higher frequency of longer IBD matches among Latinos is compatible with a recent European founder effect. After excluding within-population pairs of Latino individuals (top right), there are still more and longer IBD matches among Caribbean populations compared to Iberians (bottom right). (TIF) [file pgen.1003925.s013.tif]

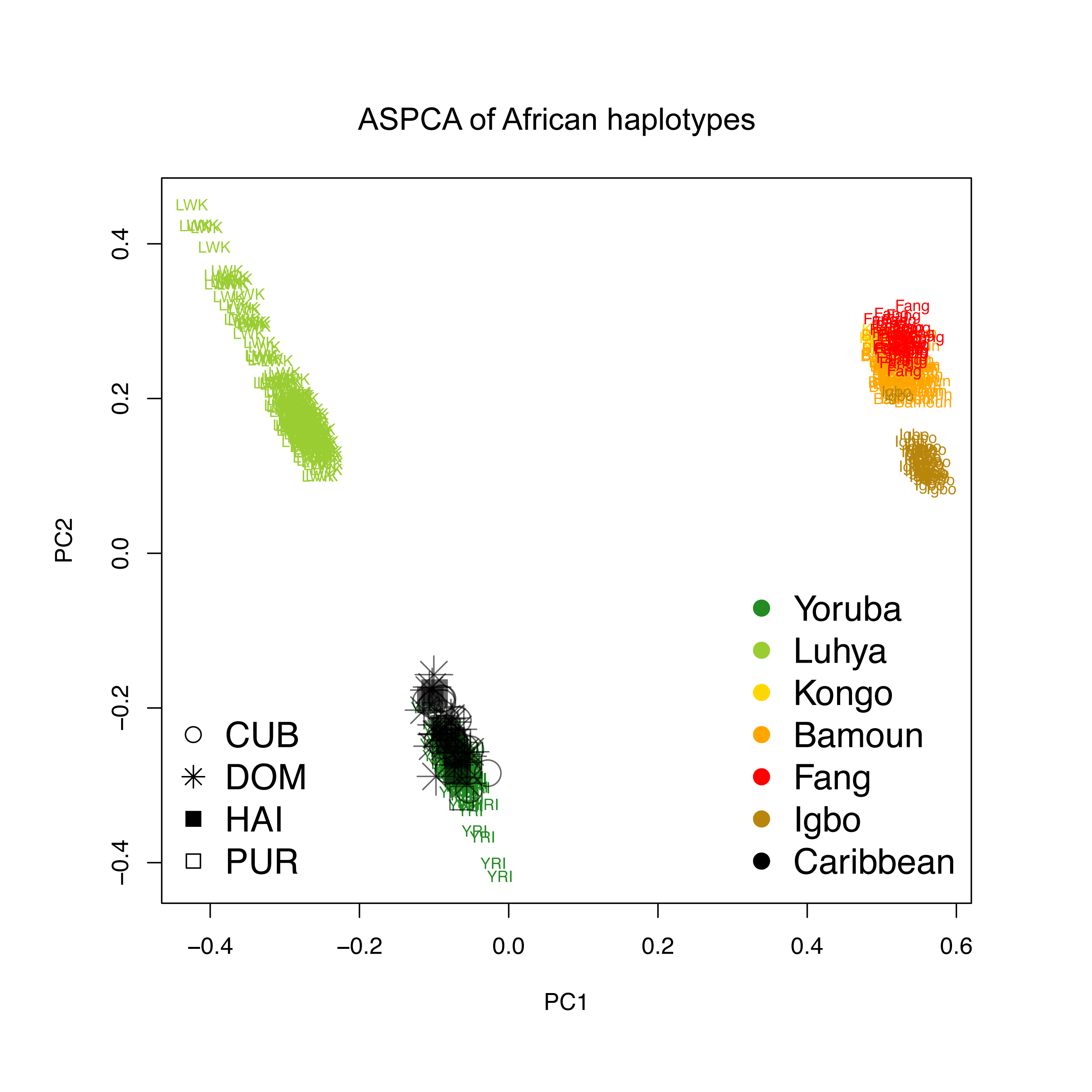

Supplement: Figure S14 — ASPCA analysis of African haplotypes derived from admixed genomes with >25% of African ancestry (black symbols) and a representative subset of African HapMap3 and other West African reference panel populations from [10]. Colombians and Hondurans excluded due to lower overall proportions of African ancestry. (TIF) [file pgen.1003925.s014.tif]

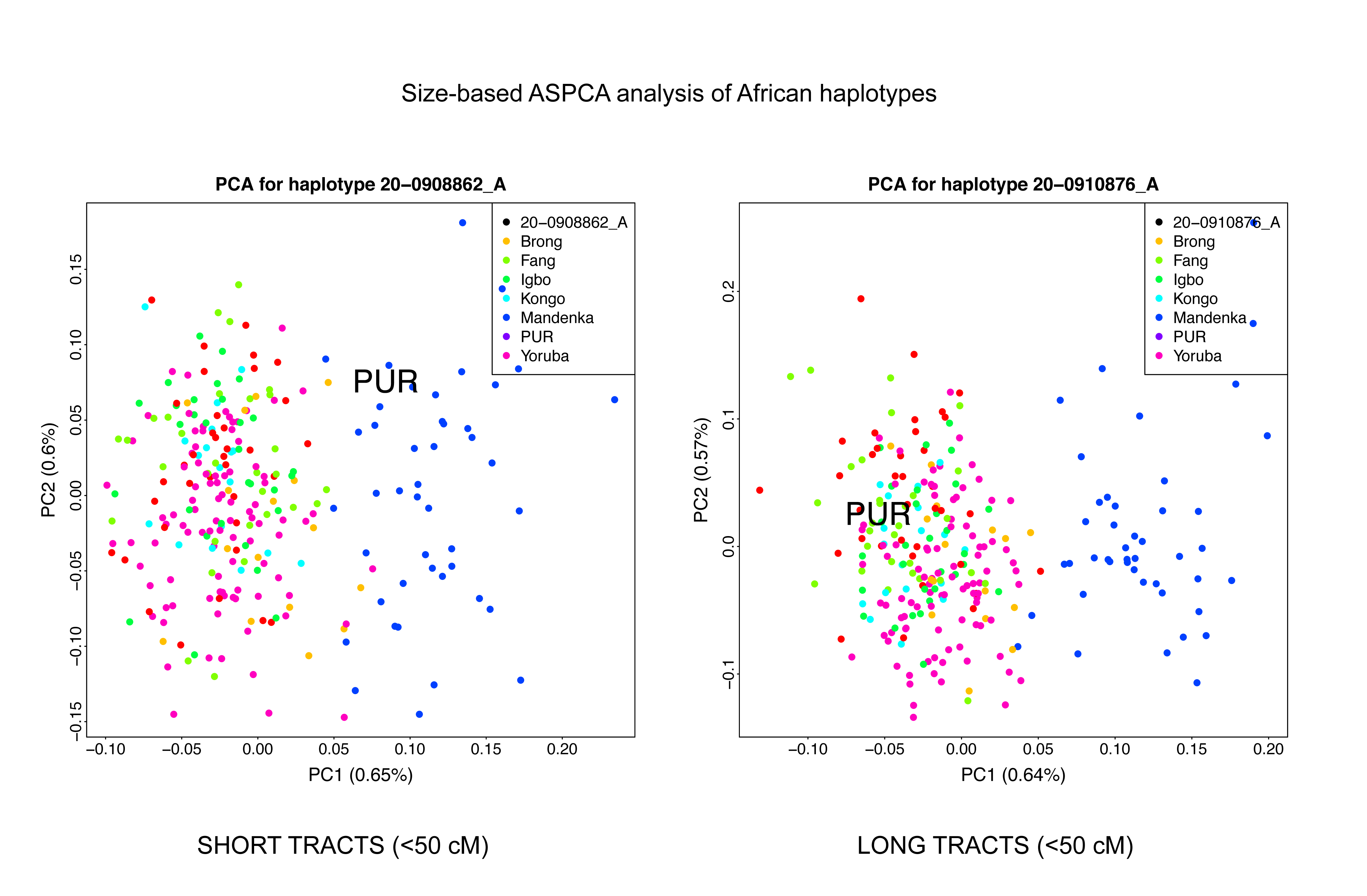

Supplement: Figure S15 — ASPCA analysis of short versus long African ancestry tracts from admixed genomes and West African reference panel populations. To exemplify our size-based ASPCA approach, the African genome of a Puerto Rican individual is displayed (denoted by PUR). Left: PUR clusters with Mandenka when only sites within short ancestry tracts (<50 cM) are considered to perform PCA. Right: a similar background distribution is obtained but the same PUR individual no longer clusters with Mandenka when considering long ancestry tracts (>50 cM). (TIF) [file pgen.1003925.s015.tif]

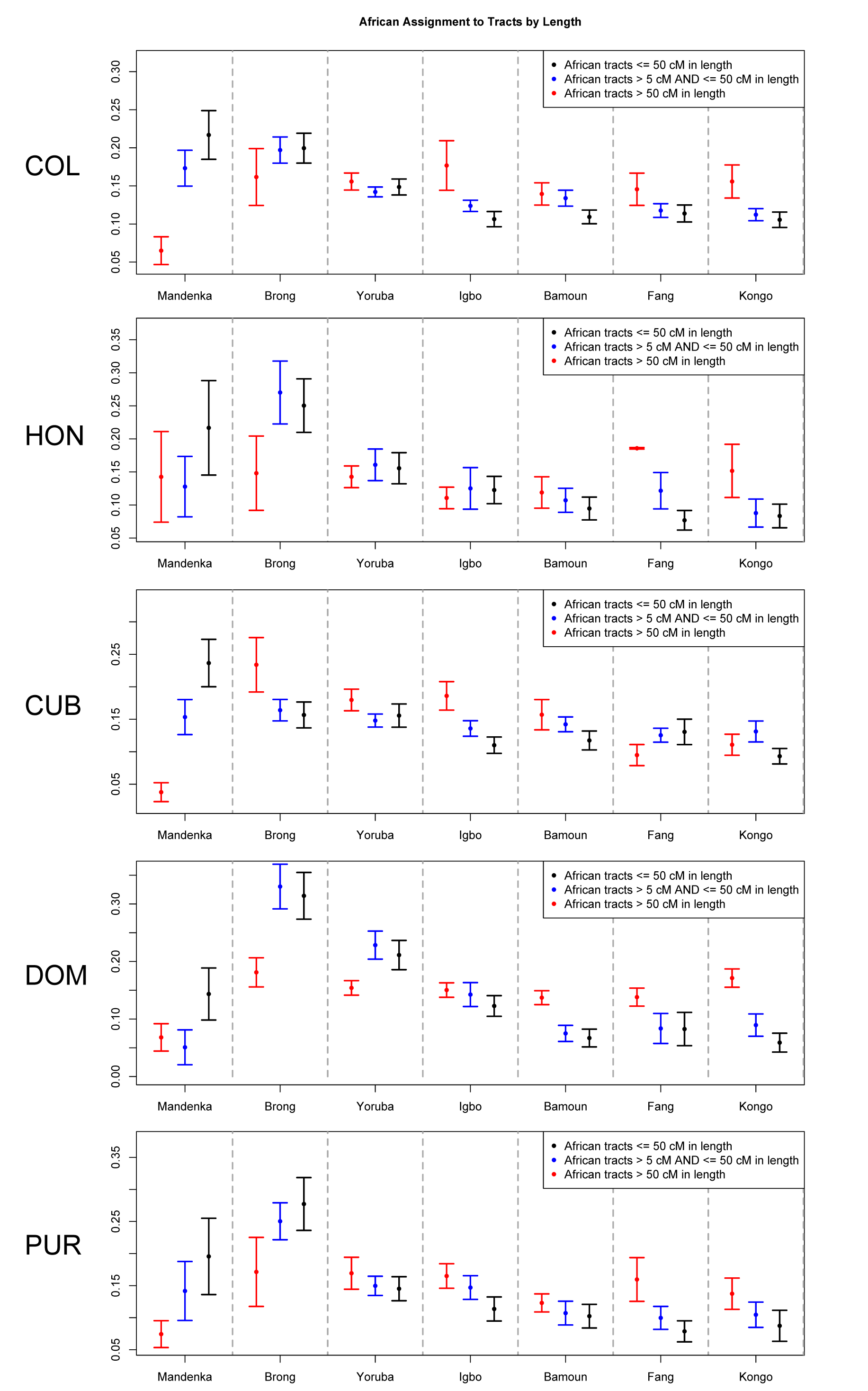

Supplement: Figure S16 — African ancestry size-based ASPCA results per population sample. Considering three different classes of ancestry tract lengths (black: short; red: long; blue: intermediate), scaled assignment probabilities are shown for each African source population. Values on the y-axis are the average probability of assignment to each potential source population across all individuals within each Latino population (see Materials and Methods for details). (TIF) [file pgen.1003925.s016.tif]
