# Supplementary material for: Reconstructing the Population Genetic History of the Caribbean
Source: PLoS Genet. 2013 Nov 14;9(11):e1003925. doi: 10.1371/journal.pgen.1003925 (PMC3828151; doi:10.1371/journal.pgen.1003925)
Supplement: Table S1 — Summary of Latino populations and assembled reference panels. (PDF) [file pgen.1003925.s017.pdf]

## Table S1

### *Summary of Latino populations and assembled reference panels*

| | Population | Pop ID | Sample size[1] | Subset in low density ADMIXTURE[2] | Subset in high density ADMIXTURE[3] | Region/country | Array platform | Study/source |
|---|---|---|---|---|---|---|---|---|
| ADMIXED LATINO | Cuban in South Florida | CUB | 80 | 52 | 52 | - | affymetrix 6.0 | *Present study* |
| | Colombian in South Florida | COL (FL) | 85 | 50 | 50 | - | affymetrix 6.0 | *Present study* |
| | Dominican in South Florida | DOM (FL) | 34 | 18 | 18 | - | affymetrix 6.0 | *Present study* |
| | Puerto Rican in South Florida | PUR (FL) | 27 | 18 | 18 | - | affymetrix 6.0 | *Present study* |
| | Honduran in South Florida | HON | 19 | 13 | 13 | - | affymetrix 6.0 | *Present study* |
| | Haitian in South Florida | HAI | 6 | 4 | 4 | - | affymetrix 6.0 | *Present study* |
| | TOTAL SOUTH FLORIDA | | 251 | 155 | 155 | | | |
| | Puerto Rican in New York | PUR (NY) | 27 | 26 | - | - | illumina 650K | Bryc et al. 2010b |
| | Dominican in New York | DOM (NY) | 27 | 26 | - | - | illumina 650K | Bryc et al. 2010b |
| | Colombian in New York | COL (NY) | 26 | 26 | - | - | illumina 650K | Bryc et al. 2010b |
| | Ecuadorian in New York | ECU | 20 | 20 | - | - | illumina 650K | Bryc et al. 2010b |
| | Colombian in Medellin | CLM (1KG) | 70 | 70 | - | - | illumina Omni 2.5 | 1000 Genomes |
| | Puerto Rican in Puerto Rico | PUR (1KG) | 70 | 70 | - | - | illumina Omni 2.5 | 1000 Genomes |
| | Mexican in Los Angeles | MXL | 80 | 48 | 48 | - | affymetrix 6.0 | HapMap3 |
| | TOTAL ADMIXED LATINOS | | 571 | 441 | 203 | | | |
| NATIVE AMERICAN | Nahua | - | 30 | - | 29 | Mesoamerica | affymetrix 500K | Mao et al. 2007 |
| | Maya | - | 25 | - | 24 | Mesoamerica | affymetrix 500K | Mao et al. 2007 |
| | Quechua | - | 25 | - | 24 | Andes | affymetrix 500K | Mao et al. 2007 |
| | Aymara | - | 25 | - | 25 | Andes | affymetrix 500K | Mao et al. 2007 |
| | Bari | - | 29 | 27 | 27 | Venezuela | affymetrix 6.0 | *Present study* |
| | Yukpa | - | 25 | 25 | 25 | Venezuela | affymetrix 6.0 | *Present study* |
| | Warao | - | 25 | 23 | 23 | Venezuela | affymetrix 6.0 | *Present study* |
| | Eskimo-Aleut | - | 23 | 23 | - | North America | illumina 650K | Reich et al. 2012 |
| | Na-Dene | - | 15 | 15 | - | North America | illumina 650K | Reich et al. 2012 |
| | Northern Amerind | - | 93 | 93 | - | North America | illumina 650K | Reich et al. 2012 |
| | Central Amerind | - | 108 | 108 | - | North America | illumina 650K | Reich et al. 2012 |
| | Chibchan-Paezan | - | 65 | 65 | - | Central America | illumina 650K | Reich et al. 2012 |
| | Andean | - | 97 | 97 | - | South America | illumina 650K | Reich et al. 2012 |
| | Ge-Pano-Carib | - | 12 | 12 | - | South America | illumina 650K | Reich et al. 2012 |
| | Equatorial-Tucanoan | - | 80 | 80 | - | South America | illumina 650K | Reich et al. 2012 |
| | TOTAL NATIVE AMERICAN[4] | | 677 | 568 | 177 | | | |
| EUROPEAN | European (North West) | EUR NW | 266 | 40 | 40 | - | affymetrix 500K | POPRES[6] |
| | European (North/Norh East) | EUR NNE | 76 | 15 | 15 | - | affymetrix 500K | POPRES |
| | European (South East) | EUR SE | 96 | 8 | 8 | - | affymetrix 500K | POPRES |
| | European (East/South East) | EUR ESE | 8 | - | - | - | affymetrix 500K | POPRES |
| | European (Central) | EUR C | 186 | 20 | 20 | - | affymetrix 500K | POPRES |
| | European (West) | EUR W | 259 | 60 | 60 | - | affymetrix 500K | POPRES |
| | European (South) | EUR S | 232 | 20 | 20 | - | affymetrix 500K | POPRES |
| | European (South West) | EUR SW | 264 | 40 | 40 | - | affymetrix 500K | POPRES |
| | TOTAL POPRES[5] | | 1387 | 203 | 203 | | | |
| | Iberian in Andalusia | IBE AND | 17 | - | - | Spain | affymetrix 6.0 | Botigue et al. 2013 |
| | Iberian in Galicia | IBE GAL | 17 | - | - | Spain | affymetrix 6.0 | Botigue et al. 2013 |
| | Iberian in the Basque Country | IBE BAS | 20 | - | - | Spain | affymetrix 6.0 | Henn et al. 2012 |
| | TOTAL EUROPEAN | | 1441 | 203 | 203 | | | |
| AFRICAN | Yoruba | YRI | 167 | 50 | 58 | West Africa | affymetrix 6.0 | HapMap3 |
| | Luhya | LWK | 90 | - | - | East Africa | affymetrix 6.0 | HapMap3 |
| | Bamoun | - | 20 | - | - | West Africa | affymetrix 500K | Bryc et al. 2010a |
| | Fang | - | 18 | - | - | West Africa | affymetrix 500K | Bryc et al. 2010a |
| | Igbo | - | 17 | - | - | West Africa | affymetrix 500K | Bryc et al. 2010a |
| | Kongo | - | 9 | - | - | West Africa | affymetrix 500K | Bryc et al. 2010a |
| | Brong | - | 8 | - | - | West Africa | affymetrix 500K | Bryc et al. 2010a |
| | Mandenka | - | 24 | - | - | West Africa | illumina 650K | HGDP |
| | TOTAL AFRICAN | | 353 | 50 | 58 | | | |
| | TOTAL | | 3042 | 1262 | 641 | | | |

[1]Total of samples included in the study. For Native American, European, and African populations, this is the maximum number of samples used to construct the reference panels (e.g., for ASPCA analyses).
[2]The low-density dataset consists of 30,860 SNPs from a representative subset of most populations with Affymetrix SNP array data merged with populations with Illumina SNP array data. Numbers vary from the initial sample size either due to QC filtering (e.g., trios offspring, cryptic related individuals, and PCA outliers) or to broadly equalize sample sizes across populations (e.g., within POPRES collection).
[3]The high-density dataset consists of 389,225 SNPs from a representative subset of most populations with Affymetrix SNP array data. Only a subset of YRI samples were included in order to focus on European and Native American sub-continental structure (other analyses, such as ASPCA, were performed focusing on sub-continental African ancestry including all West African populations).
[4]The subset of 493 Native American samples from Reich et al (2012) represent a total of 52 populations. However, for summary purposes, these are shown grouped by linguistic families as in the original publication.
[5]We restricted to 1,387 POPRES European samples with four grandparents from the same country as in Novembre et al. (2008) to ensure replication of the PCA map of Europe. Geographic groups are as in Auton et al (2009). Full details of studied populations by country are available in Novembre et al. (2008).
[6]The collections and methods for the Population Reference Sample (POPRES) are described by Nelson et al. (2008). The datasets used for the analyses described in this manuscript were obtained from dbGaP at http://www.ncbi.nlm.nih.gov/projects/gap/cgibin/study.cgi?study_id=phs000145.v1.p1 through dbGaP accession number phs000145.v1.p1.
