# Supplementary material for: Reconstructing the Population Genetic History of the Caribbean
Source: PLoS Genet. 2013 Nov 14;9(11):e1003925. doi: 10.1371/journal.pgen.1003925 (PMC3828151; doi:10.1371/journal.pgen.1003925)
Supplement: Table S2 — Correlation p-values of male vs. female ancestry. (PDF) [file pgen.1003925.s018.pdf]

Table S2

Correlation p-values of male vs. female ancestry[1]

Population	AFR	EUR	NAT
COL	0.00022	9.00E-05	1.00E-05
CUB	0.00023	0.00046	< 0.00001
DOM	0.03748	0.03355	0.01294
HAI	0.49834	0.49923	0.49835
HON	0.01407	< 0.00001	< 0.00001
PUR	0.04182	0.01473	0.01058
ALL	< 0.00001	< 0.00001	< 0.00001

[1]Significance was assessed by comparing correlation of ancestry assignments among parent pairs to 100,000 permuted male-female pairs in each population.
