# Supplementary material for: Reconstructing the Population Genetic History of the Caribbean
Source: PLoS Genet. 2013 Nov 14;9(11):e1003925. doi: 10.1371/journal.pgen.1003925 (PMC3828151; doi:10.1371/journal.pgen.1003925)
Supplement: Table S3 — FST divergences between estimated populations for K = 8 using ADMIXTURE. (PDF) [file pgen.1003925.s019.pdf]

Table S3

F_{ST} divergences between estimated populations for K=8 using ADMIXTURE

K=8	Eur-North	Yukpa	Warao	Bari	Eur-South	Eur-Latino	Amerind
Yukpa	0.274						
Warao	0.26	0.232					
Bari	0.277	0.239	0.234				
Eur-South	0.02	0.284	0.269	0.286			
Eur-Latino	0.015	0.264	0.25	0.266	0.021		
Amerind	0.165	0.133	0.118	0.136	0.174	0.158	
Yoruba	0.184	0.372	0.358	0.375	0.176	0.164	0.27
