# Supplementary material for: Reconstructing the Population Genetic History of the Caribbean
Source: PLoS Genet. 2013 Nov 14;9(11):e1003925. doi: 10.1371/journal.pgen.1003925 (PMC3828151; doi:10.1371/journal.pgen.1003925)
Supplement: Table S4 — FST divergences between estimated populations for K = 20 using ADMIXTURE. (PDF) [file pgen.1003925.s020.pdf]

Table S4

F_{ST} divergences between estimated populations for K=20 using ADMIXTURE

K=20	Eur-North	Surui	Chipewyan	Pima	Yukpa	Mixe	Inuit	Patagonia	Eur-Latino insular	Eur-South	Karitiana	Yoruba	Bari	Eur-Latino mainland	Cabecar	Andean	Tepehuan	Ticuna	Algonquin
Surui	0.296																		
Chipewyan	0.159	0.237																	
Pima	0.227	0.214	0.162																
Yukpa	0.29	0.259	0.229	0.206															
Mixe	0.19	0.157	0.121	0.089	0.151														
Inuit	0.191	0.259	0.153	0.185	0.256	0.144													
Patagonia	0.175	0.178	0.133	0.121	0.17	0.071	0.157												
Eur-Latino insular	0.037	0.291	0.165	0.225	0.287	0.188	0.193	0.176											
Eur-South	0.039	0.312	0.175	0.24	0.305	0.205	0.202	0.189	0.041										
Karitiana	0.272	0.216	0.213	0.185	0.232	0.132	0.234	0.153	0.269	0.287									
Yoruba	0.185	0.384	0.26	0.313	0.376	0.277	0.278	0.267	0.157	0.177	0.362								
Bari	0.291	0.259	0.229	0.204	0.239	0.15	0.251	0.169	0.288	0.304	0.231	0.377							
Eur-Latino mainland	0.035	0.289	0.161	0.221	0.279	0.184	0.19	0.171	0.045	0.04	0.264	0.174	0.278						
Cabecar	0.259	0.224	0.194	0.166	0.195	0.112	0.218	0.134	0.256	0.271	0.197	0.344	0.177	0.244					
Andean	0.196	0.154	0.13	0.102	0.151	0.047	0.152	0.068	0.194	0.21	0.131	0.283	0.151	0.188	0.115				
Tepehuan	0.192	0.171	0.13	0.094	0.166	0.054	0.154	0.086	0.191	0.206	0.146	0.279	0.165	0.186	0.128	0.066			
Ticuna	0.206	0.157	0.136	0.104	0.149	0.055	0.156	0.075	0.201	0.22	0.131	0.289	0.142	0.195	0.119	0.051	0.067		
Algonquin	0.188	0.281	0.168	0.206	0.275	0.164	0.21	0.177	0.195	0.201	0.253	0.294	0.271	0.19	0.237	0.171	0.173	0.177	
Warao	0.278	0.241	0.214	0.193	0.236	0.135	0.238	0.156	0.276	0.295	0.215	0.363	0.234	0.27	0.202	0.136	0.151	0.134	0.258
