# Supplementary material for: Reconstructing the Population Genetic History of the Caribbean
Source: PLoS Genet. 2013 Nov 14;9(11):e1003925. doi: 10.1371/journal.pgen.1003925 (PMC3828151; doi:10.1371/journal.pgen.1003925)
Supplement: Text S1 — Methodology of the Ancestry-Specific PCA (ASPCA) implementation. (PDF) [file pgen.1003925.s021.pdf]

Text S1. Methodology of the Ancestry-Specific PCA (ASPCA) implementation

Overview of the ASPCA method (subspace learning algorithm): The method we describe here is a close adaptation of the *subspace learning algorithm* described in [38] to haplotype data. In contrast to the standard approach, which computes all principal components, the subspace algorithm does away with the covariance matrix altogether, and computes the first d principal components, where $1 \leq d \leq n$. Specifically, given an m x n matrix of haplotypes, the algorithm seeks to obtain the decomposition $\mathbf{X} \approx \mathbf{AS}$, where $\mathbf{S}$ is a m x d matrix, and $\mathbf{A}$ is a d x n matrix containing the top *d* principal component loadings for every individual in the sample. For our purposes, we are interested in obtaining the latter to approximate PCA. In the absence of missing data, this decomposition can be obtained iteratively by gradient descent. Starting with random matrices $\mathbf{A}$ and $\mathbf{S}$, the following update rules are alternatively applied to each matrix until convergence is achieved:

$$\mathbf{A} \leftarrow \mathbf{A} + \gamma(\mathbf{X} - \mathbf{AS})\mathbf{S}^{\mathbf{T}}$$

$$\mathbf{S} \leftarrow \mathbf{S} + \gamma\mathbf{A}^{\mathbf{T}}(\mathbf{X} - \mathbf{AS})$$

where γ controls the learning rate. Note that the resulting matrices are not necessarily orthogonal. However, orthogonalization can readily be performed post-hoc. For instance, one can orthogonalize A by SVD. Letting $\mathbf{A} = \mathbf{UDV}^{\mathbf{T}}$, the orthogonalization is computed as:

$$\mathbf{A}^* = \mathbf{UV}^{\mathbf{T}}$$

The progression of the algorithm towards convergence can be followed by tracking the change in the cost function C at every iteration, where C is defined as:

$$C = \sum_{i=1}^{n}\sum_{j=1}^{m}(x_{ij} - \sum_{k=1}^{d} a_{ik}s_{kj})^2$$

Intuitively, this is the mean square error between the data matrix **X** and its estimate **AS**. Throughout the algorithm, C is expected to converge to a local optimum in a monotonically decreasing fashion.

Focusing on a specific ancestry component (introduction of missing data): Given this framework, the above equations can be readily adapted to the presence of missing data, corresponding to regions of the genome that have been masked out to enable the study of a specific ancestral component of admixture. Specifically, instead of iterating over all possible entries of the haplotype matrix, we now only focus on those that are non-missing (i.e. those determined by the ancestry deconvolution algorithm to be derived from the desired admixture component). Thus, the cost function becomes:

$$C = \sum_{(i,j)\in O} (x_{ij} - \sum_{k=1}^{d} a_{ik}s_{kj})^2$$

where O now denotes the set of all observed values in the haplotype matrix X. Concordantly, the update equations corresponding to the gradient descent algorithm become [38]:

$$a_{ik} \leftarrow a_{ik} - \gamma \frac{\partial C}{\partial a_{ik}} = a_{ik} + \gamma \sum_{j|(i,j)\in O} \sum_{k=1}^{d} (x_{ij} - a_{ik}s_{kj})^2 s_{kj}$$

$$s_{kj} \leftarrow s_{kj} - \gamma \frac{\partial C}{\partial s_{kj}} = s_{kj} + \gamma \sum_{i|(i,j)\in O} \sum_{k=1}^{d} (x_{ij} - a_{ik}s_{kj})^2 a_{ik}$$

Implementation: Our implementation of the algorithm, which we packaged into the software *PCAmask*, follows the guidelines of the seminal paper quite closely [38]. Specifically, we adapted the standard gradient descent outlined above to include a speed-up term for faster

convergence. We achieved this by multiplying the gradient by the inverse of the second order derivatives of the cost function, as described in [38]:

$$a_{ik} \leftarrow a_{ik} - \gamma \left(\frac{\partial^2 C}{\partial a_{ik}^2} \right)^{-1} \frac{\partial C}{\partial a_{ik}}$$

$$s_{kj} \leftarrow s_{kj} - \gamma \left(\frac{\partial^2 C}{\partial s_{kj}^2} \right)^{-1} \frac{\partial C}{\partial s_{kj}}$$

Finally, we followed the guidelines described in [38] to set the convergence term γ. At the beginning of the algorithm, we set $\gamma = 1$. At every iteration, γ is then updated based on the new value C_{next} of the cost function. If $C_{next} < C$, we set $\gamma' = 1.1\gamma$; otherwise, the update of **A** and **S** is rejected and $\gamma' = \gamma/2$. This approach ensures that smaller steps are taken as the process nears the local optimum.

<u>ASPCA analysis of ancestry-specific haplotypes:</u> We used *PCAmask* (described above) to perform PCA on masked genomes of admixed origin exposing haploid loci of estimated African, European, or Native American ancestry, separately. We restricted to haploid genomes with more than 25% of European or African ancestry to be considered in the analysis. Due to the relatively low Native American ancestry in many of the samples, we lowered the inclusion threshold for this ancestral component to 3%. This allowed for the maximization of samples in the analysis while limiting the introduction of statistical noise resulting from individuals with very little Native American ancestry. For each continental ancestry, a reference panel of putatively ancestral sub-continental populations was built (see Table S1). The initial analysis of Native American segments included 218 indigenous haplotypes derived from admixed samples, and 1,100 from the reference panel (i.e., all 493 individuals from [11], and 57 native Venezuelans). Aleutians, Greenlanders, native Venezuelans, and Surui were detected as the most extreme

outliers in PCA space (see Figure S7), and were thus removed from subsequent ASPCA analyses. The final analysis included a reference panel of 892 native haplotypes. For plotting, samples were pooled by two complementary systems: by linguistic families as reported in [11] (Figure S7), and by country of origin as a proxy for geography (Figure 4A). The analysis of European segments included 255 haplotypes from the admixed populations, and 2,882 from the reference panel (i.e., the subset of 1,387 European PopRes samples used in [15], plus 54 additional Iberian individuals sampled in Spain [24]. In this case we restricted to the same set of SNPs used in [15] (i.e., 192,821 sites after merging) in order to ensure the reproduction of the PCA map of Europe by Novembre et al. Finally, the analysis of African segments included 55 haplotypes from the admixed populations, and 538 from the reference panel (i.e., 205 HapMap African samples: Yoruba from Nigeria and Kenyan Luhya; as well as 64 West African individuals from [10], including Kongo, Bamoun, Fang, and Igbo). In all cases, we define the Principal Component space using the combined set of ancestry-specific Latino haplotypes plus the corresponding reference panel. The advantage of this approach (over defining the PC's using the reference panel and projecting the Latino haplotypes) is that it allows to capture components of variation that may be absent from the reference panels.
